# Early production of table olives at a mid-7th millennium BP submerged site off the Carmel coast (Israel)

**DOI:** 10.1038/s41598-020-80772-6

**Published:** 2021-01-26

**Authors:** E. Galili, D. Langgut, J. F. Terral, O. Barazani, A. Dag, L. Kolska Horwitz, I. Ogloblin Ramirez, B. Rosen, M. Weinstein-Evron, S. Chaim, E. Kremer, S. Lev-Yadun, E. Boaretto, Z. Ben-Barak-Zelas, A. Fishman

**Affiliations:** 1 Zinman Institute of Archaeology, University of Haifa, Mount Carmel, Aba Khoushy Ave. 199, Mount Carmel, 3498838 Haifa, Israel; 2 POB 180, 3033731 Atlit, Israel; 3 Leon Recanati Institute for Maritime Studies, University of Haifa, Mount Carmel, Aba Khoushy Ave. 199, Mount Carmel, 3498838 Haifa, Israel; 4 Laboratory of Archaeobotany and Ancient Environments, Institute of Archaeology, and The Steinhardt Museum of Natural History, Tel Aviv University, 6997801 Tel Aviv, Israel; 5 Institut Des Sciences de L’Evolution de Montpellier UMR 5554, Université de Montpellier, Place Eugène Bataillon - CC065, 34095 Montpellier Cedex 5, France; 6 LIA (Laboratoire International Associé) - IRP (International Research Program) EVOLEA (INEE CNRS), Montpellier, France; 7 Agricultural Research Organization, Institute of Plant Sciences, 7505101 Rishon Le Zion, Israel; 8 Agricultural Research Organization, Gilat Research Center, M.P. Negev, 85280 Gilat, Israel; 9 National Natural History Collections, Faculty of Life Science, The Hebrew University, E. Safra Campus, 91904 Jerusalem, Israel; 10 Independent Researcher, 26 Kaplanski Street, Petah Tiqva, Israel; 11 Zinman Institute of Archaeology, University of Haifa, Aba Khoushy Ave. 199, Mount Carmel, 3498838 Haifa, Israel; 12 Department of Biology and Environment, Faculty of Natural Sciences, University of Haifa-Oranim, 36006 Tiv’on, Israel; 13 D-REAMS Radiocarbon Dating Laboratory, Scientific Archaeology Unit, Weizmann Institute of Science, 7610001 Rehovot, Israel; 14 Department of Biotechnology and Food Engineering, Technion-Israel Institute of Technology, 3200003 Haifa, Israel

**Keywords:** Plant sciences, Plant domestication

## Abstract

We present here the earliest evidence for large-scale table olive production from the mid-7^th^ millennium BP inundated site of Hishuley Carmel on the northern Mediterranean coast of Israel. Olive pit size and fragmentation patterns, pollen as well as the architecture of installations associated with pits from this site, were compared to finds from the nearby and slightly earlier submerged Kfar Samir site. Results indicate that at Kfar Samir olive oil was extracted, while at Hishuley Carmel the data showed that large quantities of table olives, the oldest reported to date, were prepared. This process was most probably facilitated by the site’s proximity to the Mediterranean Sea, which served as a source of both sea water and salt required for debittering/pickling/salting the fruit, as experimentally demonstrated in this study. Comparison of pit morphometry from modern cultivars, wild-growing trees and the archaeological sites, intimates that in pit morphology the ancient pits resemble wild olives, but we cannot totally exclude the possibility that they derive from early cultivated trees. Our findings demonstrate that in this region, olive oil production may have predated table olive preparation, with each development serving as a milestone in the early exploitation of the olive.

Throughout the Mediterranean Basin, the olive tree is considered an emblematic and economically important species (e.g.,^1–3^). The domesticated form (*Olea europaea* subsp. *europaea* var. *sativa*), commonly known as *Olea europaea*, has given rise to hundreds of cultivars in different geographic areas^4,5^. Its main wild progenitor, commonly known as oleaster (*O*. *europaea* subsp. *europaea* var. *sylvestris* [Mill.] Lehr.), is a typical but a minor component of the natural Mediterranean garigue, maquis and forest landscapes. Identification of the earliest domestic olives has proved to be complex. Genetic research has demonstrated that reciprocal gene flow regularly took place between wild and domesticated types^6,7^, while oleaster plants have served as stock material onto which cultivated clones are grafted^8–10^. This might partly explain why genetic studies have reached dissimilar conclusions regarding the number of domestication events and geographic origin of *Olea* domestication^11–15^.

In the Levantine region of the Eastern Mediterranean, palynological evidence for the presence of *Olea* var. *sylvestris* goes back to the Middle Pleistocene (e.g.,^16–20^). A significant increase in *Olea* sp. pollen grain percentages during the 7th millennium BP has led researchers to conclude that olive cultivation first occurred in the southern Levant by ~ 6,500 years BP, and later dispersed to other parts of the Mediterranean Basin^21–23^. Remains of olive wood and/or olive pollen have been reported from many Late Pleistocene-Early Holocene Southern Levantine sites, including the 9th millennium BP submerged site of Atlit Yam^24^. This was followed by a significant increase in the quantities of olive pits recovered from numerous archaeological deposits in the Southern Levant dating to 7,000 to 6,500 BP^25–31^. Thus, both archaeobotanical finds and pollen data suggest that the initial cultivation of olives first took place in the Southern Levant no later than ~ 6,500 BP^23,25,31,32^.

While the earliest evidence for olive fruit processing relates to olive oil production, and dates to ~ 7,000 years ago^28^, the timing of the earliest production of table olives is currently unknown. Archaeological and written information on the consumption of table olives relates to Classical periods but the origin of the practice of debittering olives for human consumption, is as yet undated. It is assumed that in pre-Hellenistic Egypt, table olives were not available prior to Egypt's conquest of Alexander^33^. In his history of Greek foodways, Dalby^34^ wrote that the origin of preservation of olives using salt is unknown, noting that they were eaten prior to, but never during the main meal. There are written accounts that olives were part of the rations given to 5th century BC Greek mercenaries^35^, while André^36^ showed that even mythical and legendary claims for the earliest appearance of the olive in the Roman diet, date to no earlier than the 8th century BC.

The aim of this article is to present and discuss recent finds relating to table olives from a unique archaeological context in the inundated, mid-7th millennium BP site of Hishuley Carmel on the Mount Carmel coast, northern Israel (Fig. 1a,b; SI Appendix 1). The finds provide evidence for the preparation of olives for consumption, and is the earliest example known so far. Notably, they predate the historical evidence for consumption of table olives by almost four millennia.

**Figure 1:**
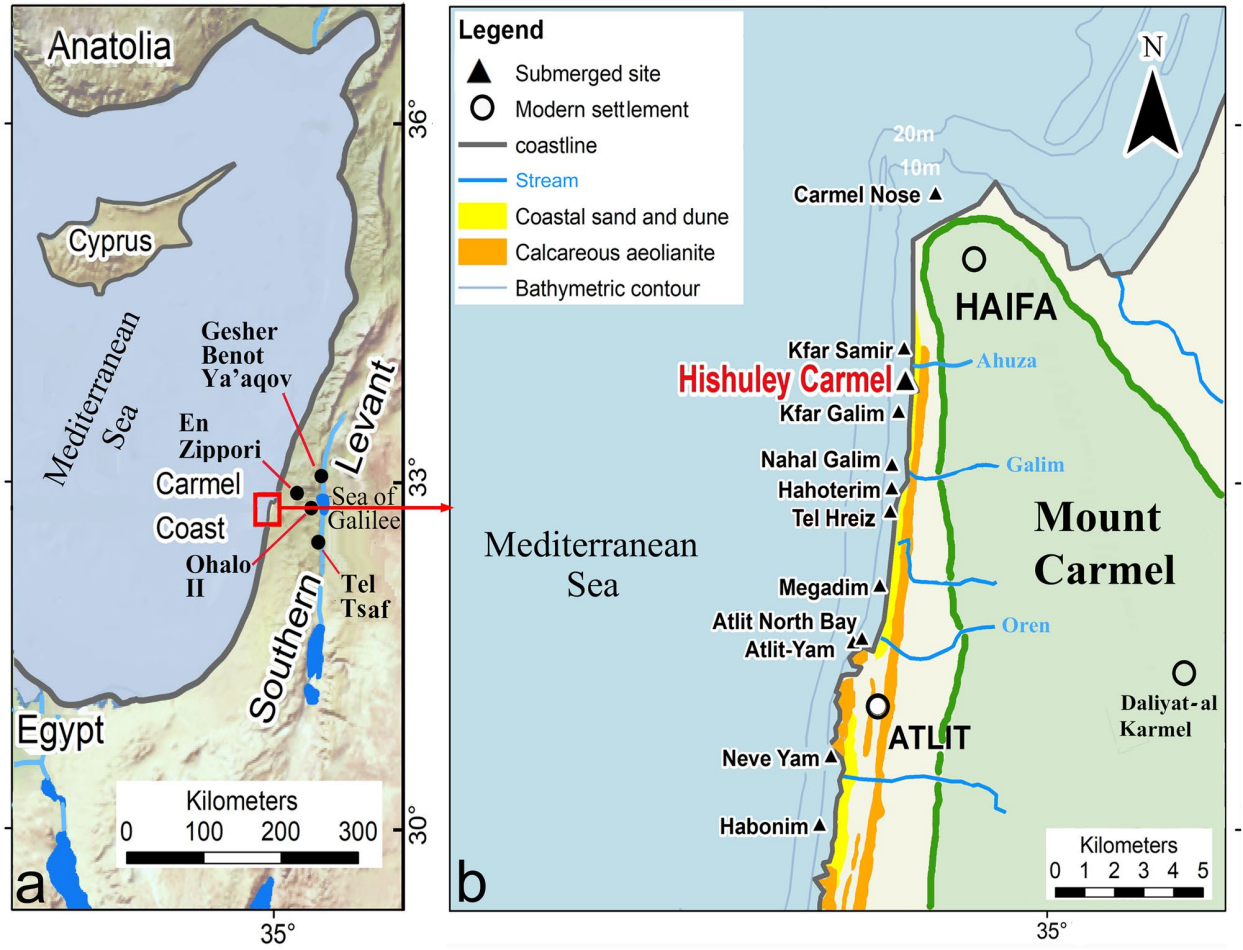
Location maps: (**a**) The Eastern Mediterranean and Southern Levant, and some sites mentioned in the text; (**b**) the Carmel coast and the submerged sites. Figures 1a, 1b were drawn by J. McCarthy after Natural Earth (https://www.naturalearthdata.com in the public domain). The two maps were modified by E. Galili using Adobe Photoshop CC 2018.

## Material and methods

Off the Carmel coast of northern Israel, 19 submerged Neolithic sites have been discovered that were inundated following post-glacial sea-level rise. The oldest site, Pre-Pottery Neolithic C (PPNC) Atlit Yam, is located 200–400 m offshore at water depth of 8–12 m, while a series of 18 more recent Late Pottery Neolithic/Chalcolithic (LPN/CH) sites (8th millennium BP) lie closer to shore in the inter-tidal and surf zones, at water depths of 0–7 m^37,38^ (see also SI Appendix 1). The sites have yielded stone-built architectural features, human burials, water wells, lithic, groundstone, bone, woven and wood objects and artefacts as well as faunal and botanical remains. The excellent conservation of the organic remains and repertoire of many unique and well-preserved architectural features (e.g. water wells, a sea wall, megaliths, cist graves)^37^, make these sites unique and of great importance for Neolithic research in the region. Some of the sites were sedentary villages while others were temporary occupations, but all were engaged in a range of subsistence activities involving fishing and agro-pastoralism.

**Figure 2:**
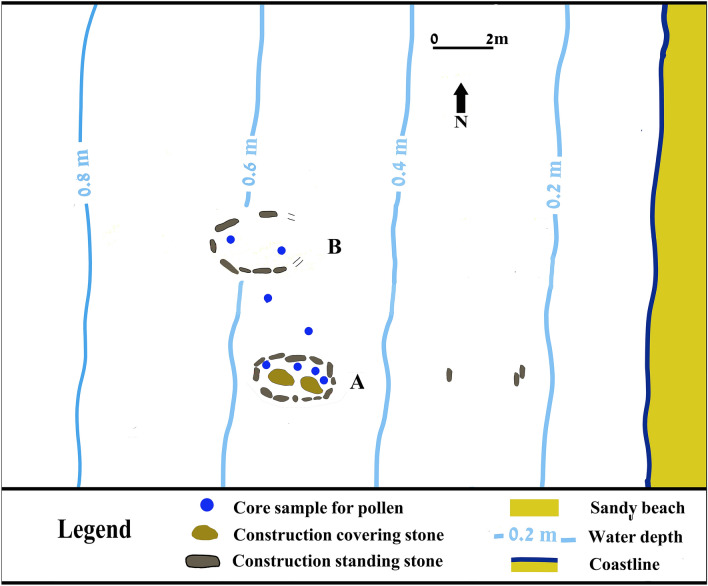
The site plan and layout of Structures A, B containing the olives. Water depth refers to the mean sea level during 2.2.2018, measured from the water surface to the clay paleosol surface (Drawing Ehud Galili).

**Figure 3:**
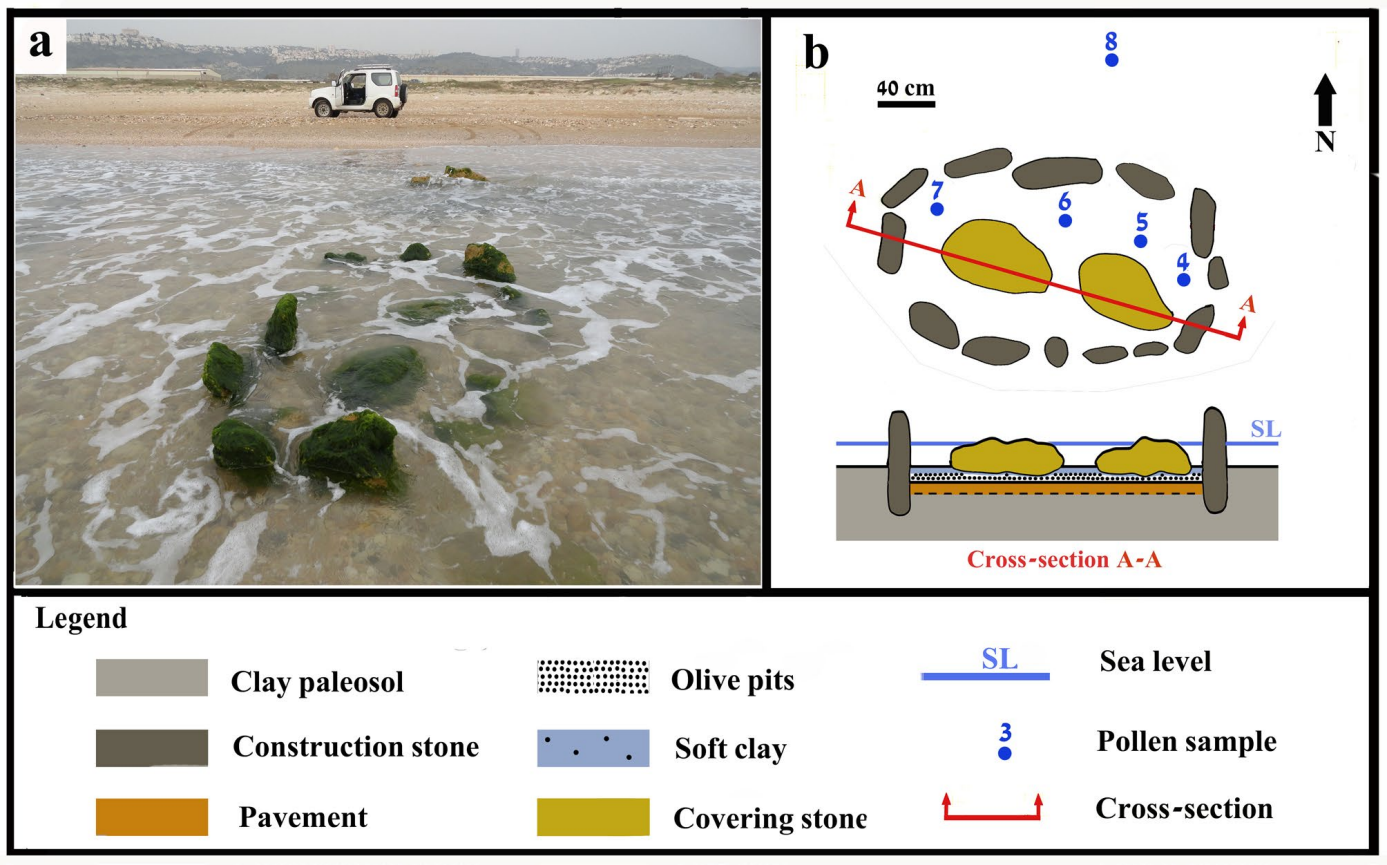
(**a**) Structure A as uncovered during 2011; (**b**) Plan and cross-section of Structure A (SL refers to the lowest tide during the drawing of the structure on the 2.2.2018 at 5 PM) (Photos and drawing Ehud Galili).

**Figure 4:**
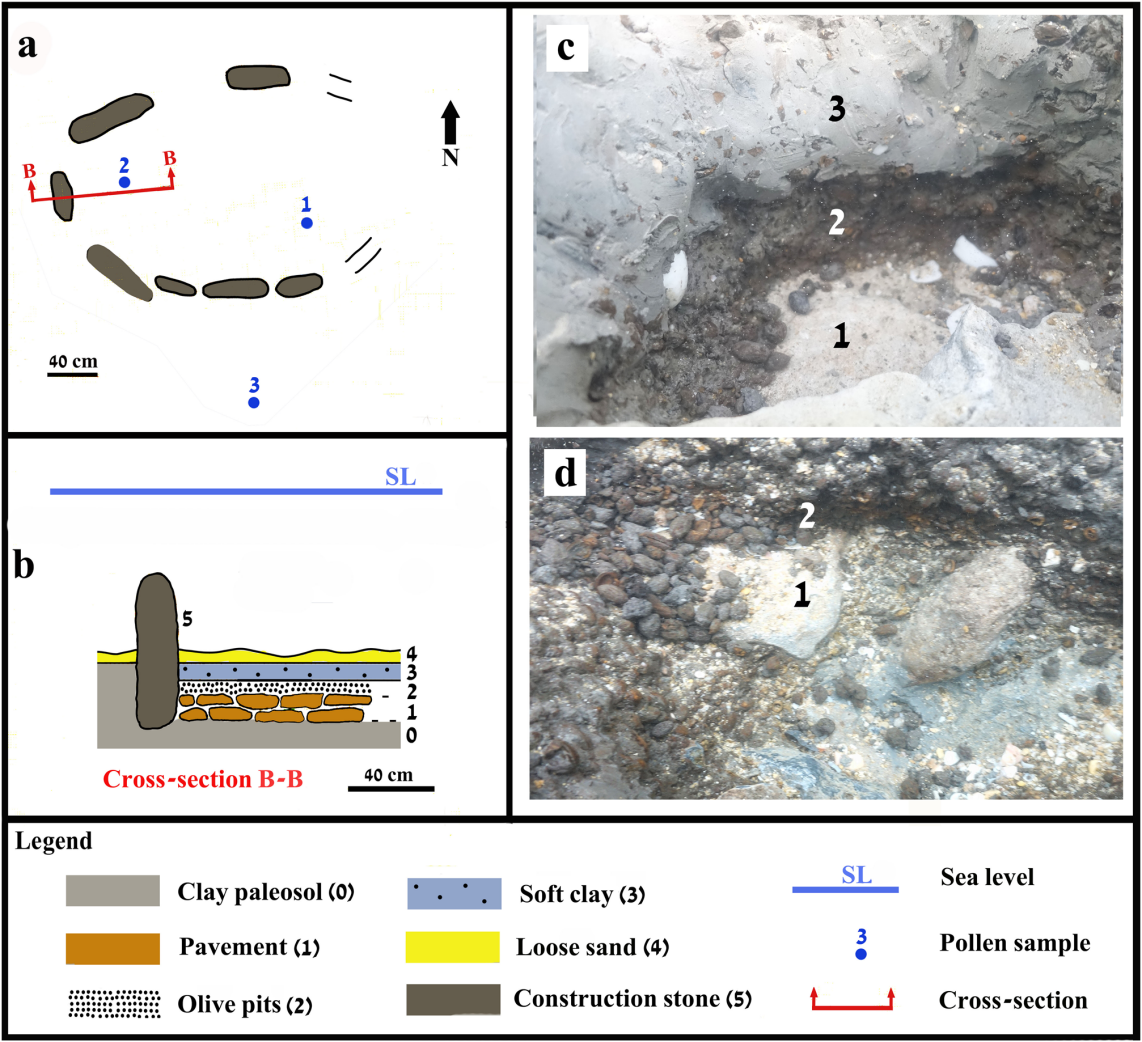
(**a**) Plan of Structure B; (**b**) Enlarged cross-section of Structure B and its layers (0–4); (**c**) Pavement (marked as 1) overlain by olive pits (marked as 2) overlain by grey, soft clay (marked as 3); (**d**) A close-up of the pavement (marked as 1) overlain by olive pits (marked as 2) (SL refers to the high tide during 13. 3. 2018, at 1.30 PM) (Photos and drawing Ehud Galili).

Underwater surveys conducted in 2011 at one of these submerged sites, Hishuley Carmel, revealed a small elliptical structure constructed of upright stone slabs and stone pavement (henceforth Structure A, Figs. 2, 3) adjacent to which were numerous olive pits^39^. Subsequently, a second similar-shaped installation of roughly the same proportions and also built of upright stone slabs, Structure B, was found 3 m to the north (Figs. 2, 4). Three further sets of standing stones, some 2.6 m apart, were found ~ 2.5–4 m east of Structure A, and probably represent eroded remains of a third structure. In order to understand the function of the structures, we examined whether there had been any a priori selection of stone used in their construction due to their natural qualities, such as texture, porosity, strength etc. To facilitate mineralogical identification, samples were taken of 12 stones from Structures A and B, and two from the paving in Structure A (SI Appendix 2). In addition, thousands of olive pits were collected from the fill inside the two structures for dating (SI Appendixes 1, 3a–c) and for examination (SI Appendix 4). Aside from the two structures containing olive pits, four additional, round installations (up to 1.5 m in diameter) made of undressed stones and located ~ 80 m offshore at 3 m depth were identified at Hishuley Carmel (SI Appendix 1: Fig. 1)**.** These structures, perhaps the upper part of water wells or storage pits, are typical of the inundated LPN/Ch sites off the Carmel coast. No other features were recovered near the structures.

The fragmentation patterns and morphometry of the Hishuley Carmel olive pits were compared to those from the slightly earlier and neighboring inundated site of Kfar Samir, where previously early large-scale olive-oil production was identified. At Kfar Samir, high concentrations of crushed olive pits and pulp and few whole pits were recovered from an unlined round pit, dug directly into the clay paleosol, which had unworked pebbles paving at its base. The highly fragmented olive pits and the high frequency of olive pollen in the associated sediments resembled that of olive-oil extraction waste (locally termed *jift* in Arabic or *gefet* in Hebrew) recovered from a modern olive-oil processing plant on Mount Carmel (SI Appendix 5). Additionally, several large stone basins found near the pit were thought to have been used for crushing olives. Woven basket-like items recovered from another excavated pit at the site resemble traditional *akals* or olive oil strainers^37^, supporting the exploitation of olives for oil at this site.

**Figure 5:**
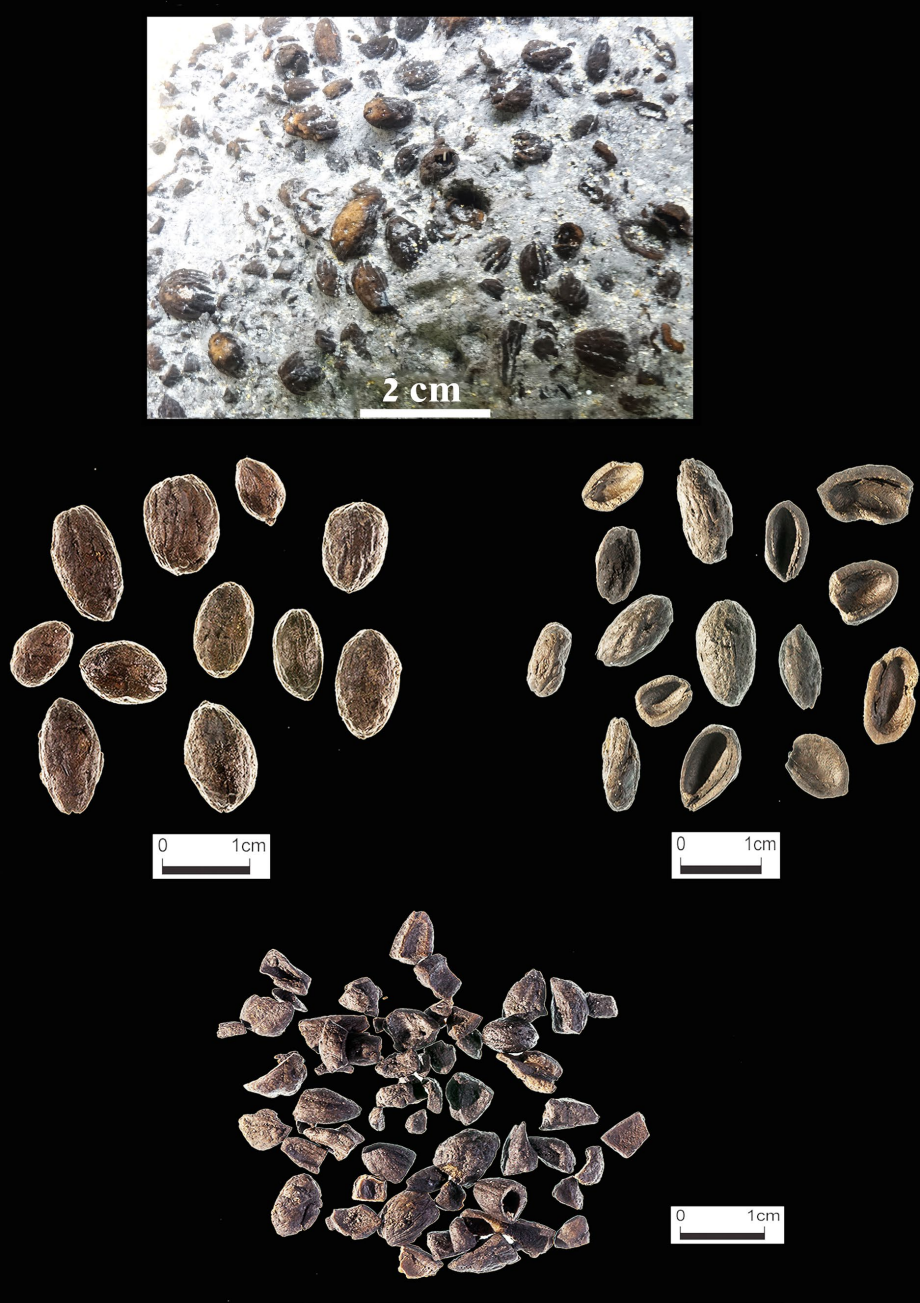
Olive pits from Hishuley Carmel: as found inside the Structures (top); whole (center left); whole and halves (center right); fragments (bottom) (Photos: Ehud Galili-top photo, Sasha Flit-center and bottom).

**Figure 6:**
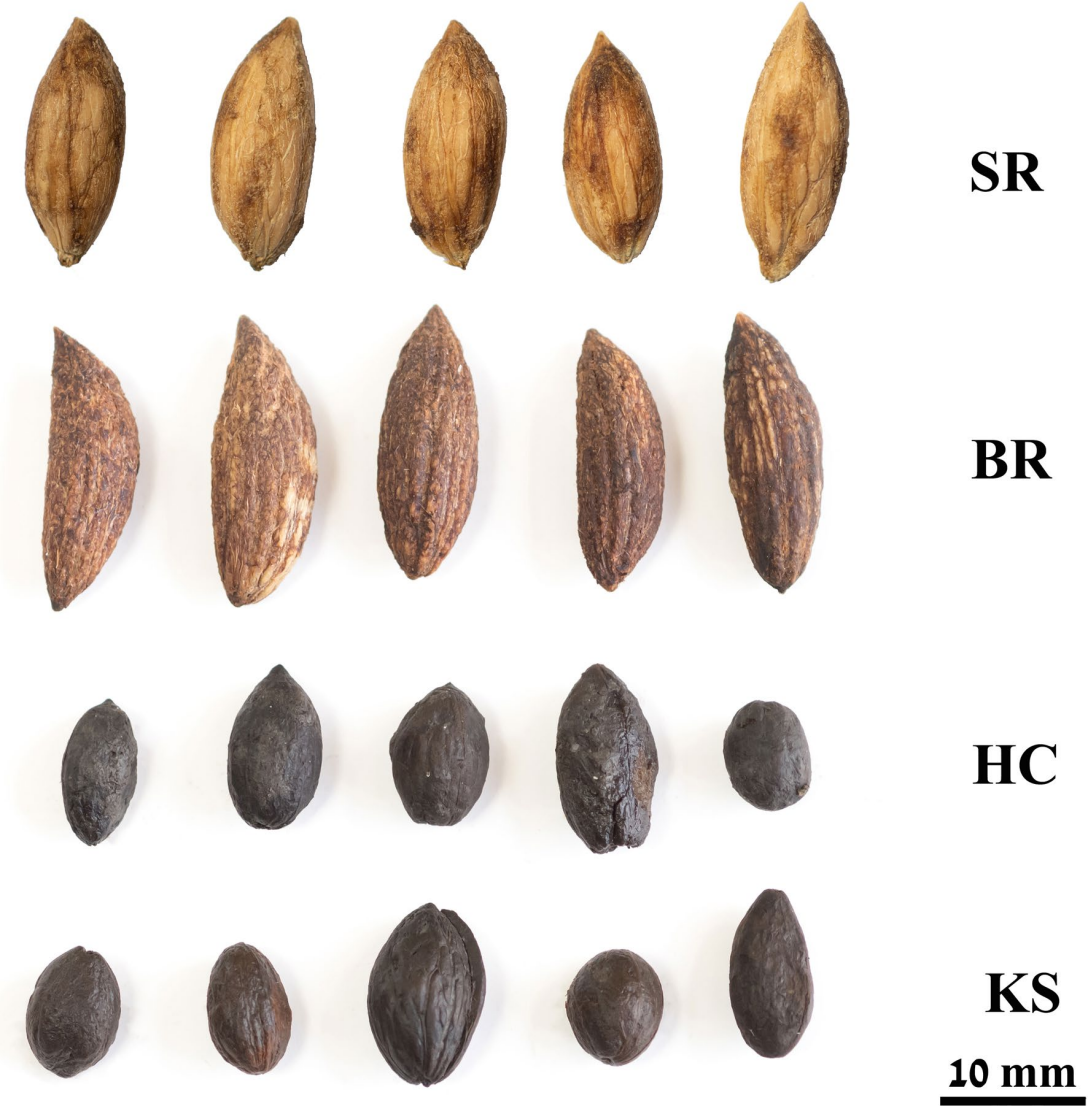
Representative olive pits of the reference cultivar Barnea (BR) and Souri (SR) and ancient pits from Hishuley Carmel (HC) and Kfar Samir (KS), showing the uniformity of the cultivars vs. variability in the structure and size of the archaeological olive pits (Photos: top lines-T. Nachshon-Dag,-bottom lines-O. Barazani).

The two archaeological olive-pit assemblages (Hishuley Carmel, and Kfar Samir) were compared in order to assess if they had served the same purpose. Fragmentation patterns were examined on a sample of 2,000 pits from both archaeological sites (Fig. 5). The pits were manually sorted and separated into: whole, halved (broken along the longitudinal suture line) and fragmented (Fig. 6). The sorted pit fractions were weighed and counted. The net weight of the whole and halved pits was calculated to compare it to the fragmented pits.

**Figure 7:**
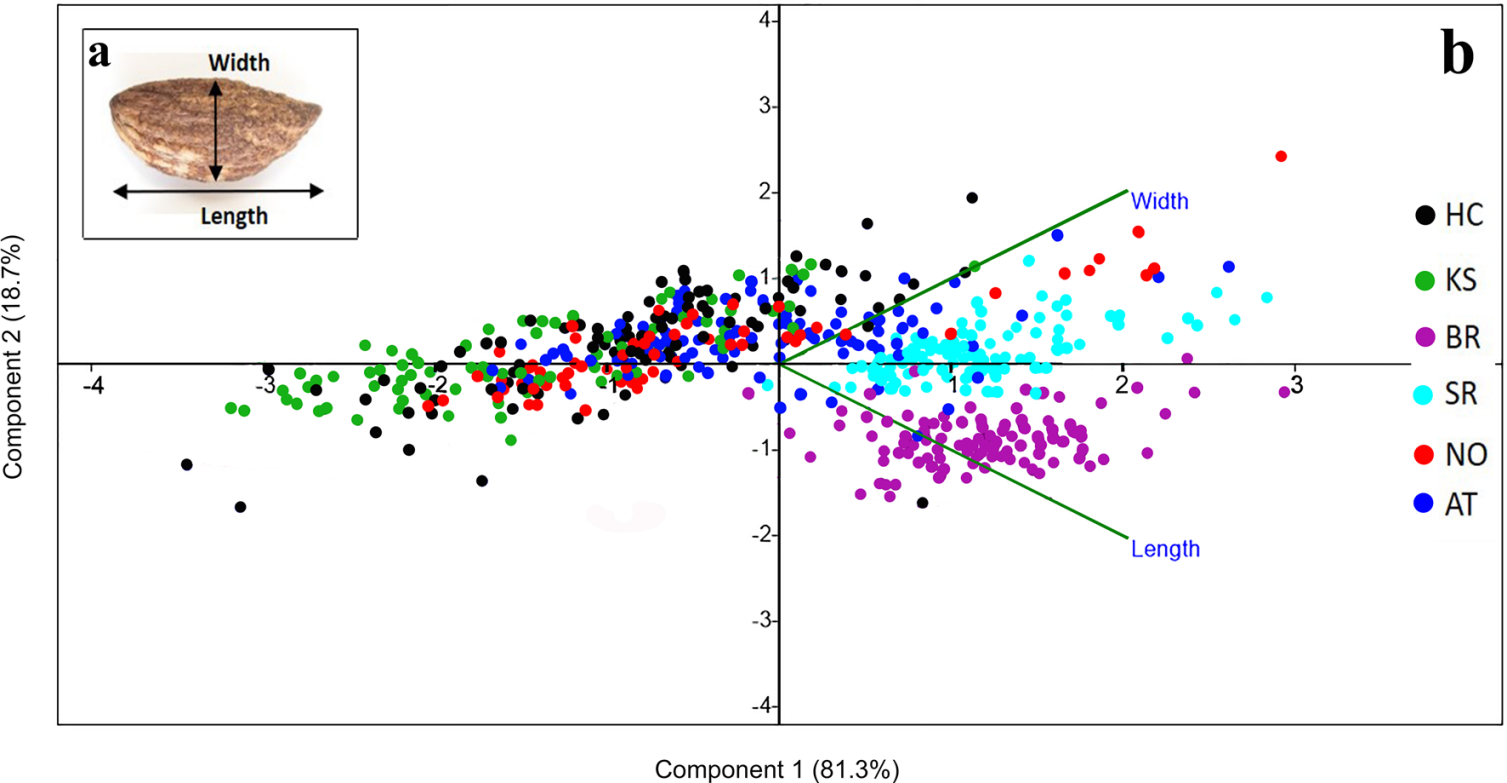
(**a**) Olive pit measurements taken. (**b**) Results of a principal component analysis (PCA) of the quantitative morphological traits: black Hishuley Carmel (HC) N = 100, green Kfar Samir (KS) N = 84, purple—Barnea (BR) N = 100, light blue—Souri (SR) N = 100, red—Nahal Oren (NO) N = 89, and dark blue—Atlit (AT) N = 60. The component 1 axis explains 81.3% of the total variation, the component 2 axis 18.7%.

The size of the Hishuley Carmel and Kfar Samir olive pits was compared to those of modern wild/feral olive trees from Atlit and Nahal Oren on Mount Carmel and two local cultivars (Fig. 6). For the metric analysis we examined 85 and 100 whole pits from Kfar Samir and Hishuley Carmel, respectively, 100 whole pits each from two local cultivars—Barnea and Souri, and 10 pits each from naturally-growing trees in the region of Mount Carmel—six trees at Atlit (34° 56′ 32.2′′ E, 32° 41′ 34.37′′ N), and nine trees at Nahal Oren (34° 58′ 39.31′′ E, 32° 42′ 48.85′′ N) (Figs. 1b, 7).

**Figure 8:**
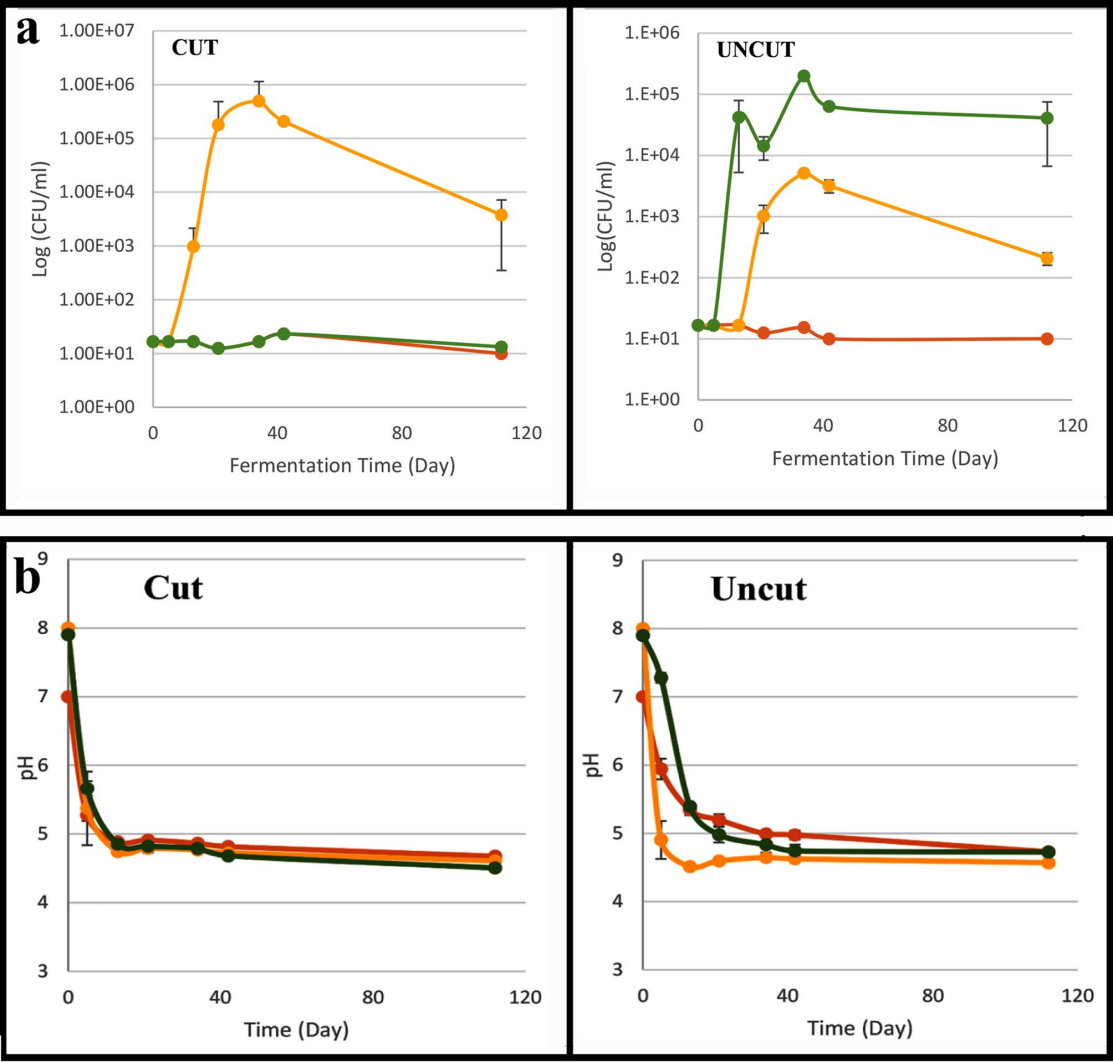

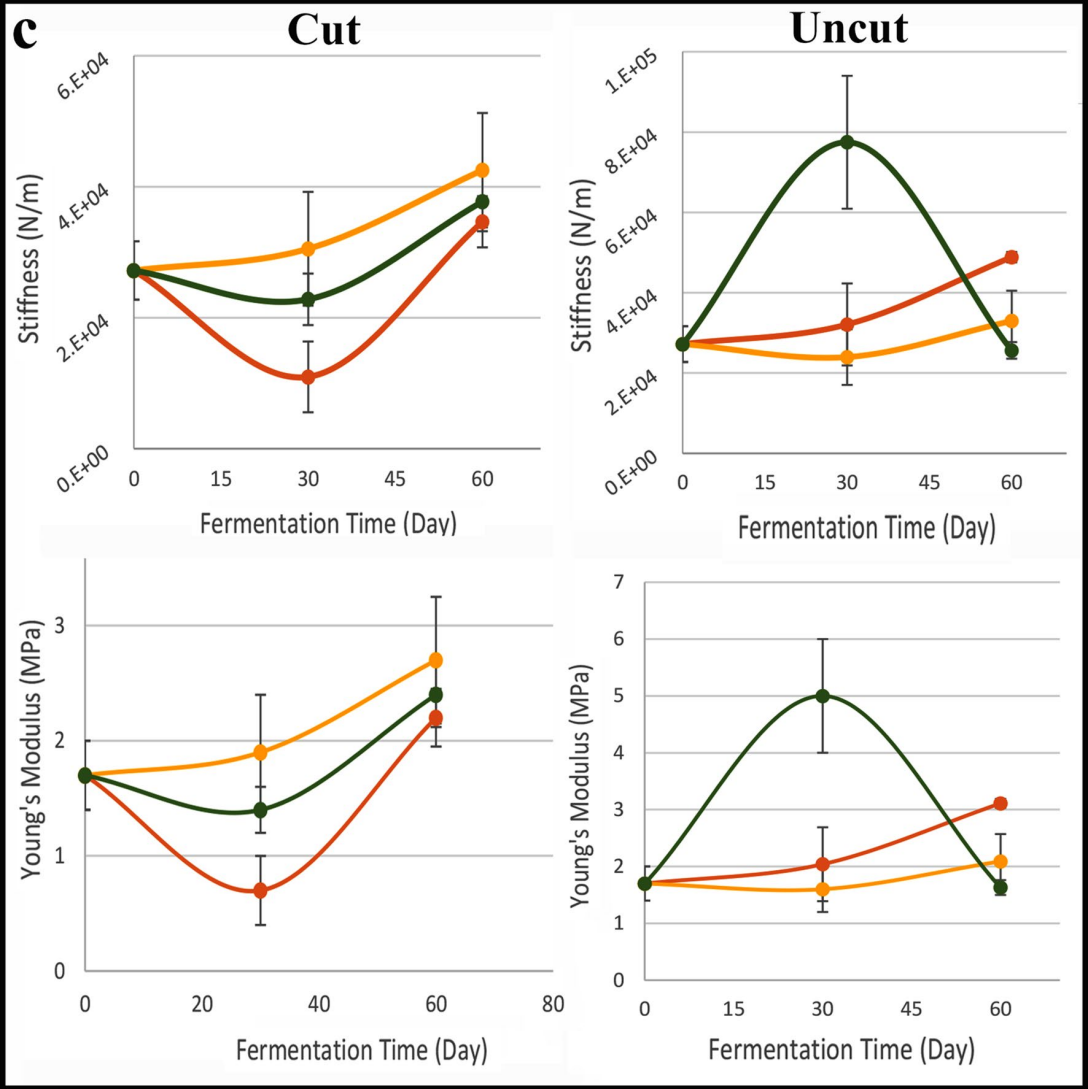
Results of experiments on different fermentation conditions. Red—Tap water + 11% NaCl; Orange—Seawater; Green—Seawater + 8% sea salt. Each point in the graphs indicates the average of three independent repetitions (N = 3). The error bars indicate the standard deviation (SD). When SD is smaller than the symbol, it is not visible in the graph: (**a**) Growth curve of total aerobic bacteria presented as log (CFU/ml) versus fermentation time. (**b**) Changes in pH values during 112 days of fermentation. As expected, the pH values decreased during fermentation. The accelerated decline was observed in fermentation with seawater brine and uncut olives. (**c**) The alteration in Young’s Modulus and Stiffness values during 60 days of fermentation.

There is extensive historical evidence for the use of sea water in curing olives [Palladius and Pliny, cited in ^36^], and it is traditionally still used in some Mediterranean regions^36,47^. Hishuley Carmel and Kfar Samir were both coastal settlements with easy access to salt and to sea water. This spurred us to conduct experiments on the suitability of sea water for olive curing and/or storage. Fermentation experiments using sea water and sea salt were undertaken to test the effectiveness of this medium in pickling olives. Three different fermentation treatments were followed using 600 Suri olives (200 per treatment) with some olives slit and others left whole (Fig. 8). Bacterial counts, pH and changes in olive flesh hardness (puncture test) were monitored for each treatment (SI Appendix 6). Experiment 1: olives fermented using sea water (~ 3% salt) (Fig. 8 in yellow); Experiment 2: olives fermented using sea water + 8% sea salt (final salt concentration ~ 11%) (Fig. 8 in green); Experiment 3: olives fermented using tap water + 11% NaCl (table salt) as a control (Fig. 8 in orange). Additionally, a pilot study of dry salting of naturally growing olives, picked on the neighbouring Atlit Ridge, was undertaken using local traditional methods (SI Appendix 6).

To further elucidate the function of the Hishuley Carmel structures, pollen analysis was undertaken of undisturbed sediments between Structures A and B, as well as samples from within each structure (SI Appendix 1). The sediments, that were collected in cores, were fully processed using standard palynological techniques and pollen grains were identified using a comparative reference collection and relevant atlases and reports (SI Appendix 5). The results were compared to the pollen assemblage from the olive-oil extraction waste at Kfar Samir, modern waste from olive-oil extraction, and natural pollen assemblages from fossil clay palaeosols from Kfar Samir and Hishuley Carmel sites (SI Appendix 5).

## Results

### The structures

When first documented, Structure A was 2.55 m long and 1.5 m wide, composed of 13 upright stones (35–55 × 40–60 × 10–15 cm) inserted into the clay paleosol (Fig. 3). They protruded up to 60 cm above it, with their tips ~ 20–30 cm above sea level at low-tide (The low sea on 2.2.2018, the day of the measurements, at 17:07 pm, was 0.045 cm above the Israel Land Survey Datum, henceforth ILSD). During high tide, these stones are submerged up to 30 cm (the high tide during 11:16 am on 2.2.2018, the day of the measurements was 0.35 m above ILSD).

Two stones (60 × 50 × 40 cm) covered the structure's fill. The bottom was paved with two layers of stone slabs (10–20 × 10–15 × 5–10 cm). The structure’s fill was ~ 15–25 cm thick, comprising two layers of soft grey clay mixed with thousands of olive pits (Fig. 3b). Structure B comprised seven upright stones (25–70 × 30–70 × 10–15 cm) (Fig. 4). The surviving length of the structure was ~ 220 cm and its width 190 cm, though originally it was probably ~ 260 cm long. The upright stones were embedded in the clay paleosol protruding up to 50 cm above it. The stones were submerged 30–70 cm during high tide. During the day of the measurements (13.3.2018) the high tide was 0.3 m above ILSD. Two layers of overlapping stones made up the bottom paving (Fig. 4b, c). The fill was ~ 15–20 cm thick and comprised of a dense layer of olive pits, overlain by grey soft clay with fewer pits.

Eight of the standing stones used to build the installations were *kurkar* (calcareous-cemented quartz sandstone of aeolian origin), two each of limestone and beachrock. One of the paving stones is *kurkar* and one of limestone (SI Appendix 2). All are local stones occurring within a few hundred meters of the site. *Kurkar* was probably quarried on the coastal ridge, limestone originated from Mount Carmel, or from the nearby Ahuza and Galim streams. The beachrock used was identified as belonging to MIS 5e (dated to ~ 125,000–120,000 years BP) and occurs in several locations on the northern Carmel coast^40^.

The two structures reflect a high degree of investment in their construction. Although they were built from a variety of local stones that apparently had not been chosen for a specific natural quality, they seem to have been selected for their shape (flatness) and similar size. Moreover, the dimensions of the installations are similar and they had both been paved with stones and probabely had cover-stones. These factors imply that the structures were constructed with care to protect their contents, even though they were not air or watertight.

Olive pits from the two structures at Hishuley Carmel were dated by radiocarbon (for details see SI Appendices 3a–c). Together, the dates place the oval structures in the mid-7th millennium BP (6,656–6,450 BP), i.e., the Middle Chalcolithic period. This makes them slightly later than the Kfar Samir olive-oil production assemblage, which was radiocarbon dated to ca. 7,000 BP (SI Appendix 3c: Fig. 1)^37^^.^

### Olive pit fragmentation

The degree of fragmentation of the Kfar Samir and Hishuley Carmel olive pit assemblages was compared (SI Appendix 4). Both methods of data calculation (by pit weight and by counts of the absolute number of pits) gave similar results for the estimated minimum number of individual pits, the percentage of whole pits and size of the fragments (Table 1). The sample of 3,113 olive pits from Hishuley Carmel was composed of 48.0% intact pits and 52.0% fragmented while in the 5,106 pits sampled from Kfar Samir, only 15.6% were intact (both whole and halved) and 84.4% were fragmented. For fragmentation pattern 2,000 pit fragments from each of the two sites were separated out and weighed. The Hishuley Carmel fragments weigh together 46.64 g—an average of 23 mg per single fragment, while the same number of fragments at Kfar Samir weighs 18.49 g—an average of 9.2 mg per single fragment. Thus, the olive pits from Kfar Samir are significantly more fragmented than those from Hishuley Carmel (SI Appendix 4).

**Table 1:** Characterization and fragmentation patterns of olive-pit assemblages from Hishuley Carmel and Kfar Samir.

Morphology of pits	Hishuley Carmel					Kfar Samir				
	Gross weight gr	Net weight gr	% of net weight	Intact pits (count or equivalent in absolute numbers)	% of total count	Gross weight gr	Net weight gr	% of total net weight	Intact pits (count or equivalent in absolute numbers)	% of total count
Whole pits	396.2	341.7	44.1	1,363	43.8	174.6	128.3	12.7	661	12.9
Halved pits	30.0	30.0	3.9	122	3.9	29.6	29.6	2.9	144	2.8
Fragments	403.0	403.0	52.0	1,628	52.3	851.5	851.5	84.4	4,301	84.2
Total	829.2	774.7	100	3,113	100	1,055.7	1,009.4	100	5,106	100

### Pit biometry

A principal component analysis (PCA) was used to visualize variation in the metric traits amongst the pits^41^. The results showed much greater size variation among the ancient olive pits of Kfar Samir, Hishuley Carmel and the living wild/feral trees of Atlit and Nahal Oren *versus* the reference cultivars, which are grouped closely together (Fig. 7). Furthermore, ANOVA test showed significant differences among the six groups in pit length (F = 425.138, *P* < 0.0001) and width (F = 49.514, *P* < 0.0001).

### Experiment of olive fermentation in sea water

When picked, green olives are bitter and have to be cured before human consumption, with pickling in brine the most common method^42,43^. During debittering, in local traditional societies, olives are soaked in salt water that is changed daily for up to ten days. Afterward, they are soaked in a 10–12% salt solution to accomplish the pickling process. Lactic acid bacteria (LAB) are essential for table olive processing, since they lower the pH and allow long-term preservation. They are typically active in salt concentrations of < 5%, and for long-term storage in up to 8% concentrations of salt^44,45^. Locally, the salt concentration in the brine used for curing green olives is determined by placing a raw chicken egg in water, and adding salt until the egg floats^46^. This practice produces brine with ~ 10% salt, ensuring a long period of conservation (usually up to a year but sometime even longer). The pickled olives are already edible after 4–6 weeks^46^.

It is important to note that fermentation of table olives is not a fully predictable process. The microbiota of olives differs by olive source and cultivar and by method of fermentation (with or without alkali treatment etc.; 48, 49). In our experiments using sea water and sea salt (SI Appendix 6), with the progress of the fermentation the enterobacteria were eliminated (Fig. 8a) and pH declined (Fig. 8b). The observed bacterial counts during fermentation with sea water were higher than in the control (tap water + 11% NaCl) (Fig. 8a). Probably, the lower salt concentration enabled the bacteria to thrive. The uncut olives with sea water + 8% NaCl had significantly higher bacterial counts than the sample of cut olives kept in the same brine (Table 2, Fig. 8a). In all fermentation conditions, we obtained similar yeast counts (Table 2). According to Heperkan^48^, yeast plays a significant role in fermentation of olives together with LAB. Similar to our results, in a previous study^50^, LAB were not observed in some types of natural green olives, possibly since LAB are moderately and indirectly inhibited due to the presence of phenolic compounds, therefore, yeasts dominated. The counts of viable yeast that were reported in the literature^48,51^ were similar to those found by us.

**Table 2:** Average yeast and mould counts along the fermentation in three different conditions. Values are mean log (CFU/ml).

	Sample	Time 0	After 34 days	After 42 days	After 112 days
Uncut olives	Tap water + 11% NaCl	1 >	4.47 ± 0.01	5.6 ± 0.1	4.2 ± 0.5
	Seawater	1 >	4.9 ± 0.2	4.91 ± 0.09	4.0 ± 0.2
	Seawater + 8% sea salt	1 >	6.0 ± 0.5	6.5 ± 0.2	6.2 ± 0.5
Cut olives	Tap water + 11% NaCl	1 >	4.7 ± 0.01	5.6 ± 0.2	4.9 ± 0.6
	Seawater	1 >	4.9 ± 0.6	5.2 ± 0.9	4.1 ± 0.1
	Seawater + 8% sea salt	1 >	2.4 ± 0.5	4.8 ± 0.8	4.6 ± 0.5

In a puncture test conducted after 30 days of fermentation, no clear trend was observed in olive flesh hardness. There was no correlation between brine type and cutting and puncture test parameters (Young's Modulus, Stiffness) compared to the initial values before fermentation (Fig. 8c; SI Appendix 6). Moreover, after two months, the values were similar across all treatments. Since the use of sea water in olive fermentation gave similar values as other brines, our experiment has shown that olives can successfully be fermented using sea water. Likewise, no differences were reported by Koprivnjak et al*.*^47^ for storage of olives in brine *versus* sea water, before oil production.

### Pickling olives with sea salt

An alternative to pickling olives with sea water prior to consumption, is to produce dry-salted table olives. This involves washing the fruit with water, mixing the washed fruit with large amounts of dry salt, followed by storing the mixture in wooden boxes or in baskets made of natural fibres or in cloth bags. Ramirez et al*.*^52^ undertook an experiment of dehydrated olive preparation by using dry-salt. They mixed the olives with salt and this mixture was kept at ambient temperature in a drum with a drainage point at the base for liquid run-off. LAB were not found and yeasts were the main microorganisms found on the olives’ surface. The dry‐salting process, which in this experiment took 42 to 48 days, also resulted in debittering the olives. However, a high concentration of salt has to be mixed with the olive juice in order to ensure the chemical and microbial stability of the dehydrated olive product.

We undertook a pilot experiment on 800 g of naturally growing olives picked during January 2020 on the Atlit Kurkar ridge using sea salt (SI Appendix 7). Boiling water was poured on the olives. They were then covered for 10 days with a 1 cm layer of thick, coarse sea salt, which had been collected in natural pools on the Carmel coast, and after 10 days the olives were mixed with the sea salt. The olives were edible after 20 days. Mediterranean Sea water contains ~ 3.5% salt^53^. Thus, for salting of dehydrated olives, sufficient quantities of salt could have been collected from small coastal pools fed with wave splash on the rocky coasts near the Hishuley Carmel site^54^.

### Pollen analysis

Samples from both structures contained well-preserved pollen, but the olive pollen percentage was very low in both. These two samples vary in ratios and component composition, including of arboreal pollen, which may result from the olives having been picked at different locations. Yet, both samples indicate a typical Mediterranean vegetation as found today in the Mount Carmel region, and on the coastal plain in the past, comprising mainly evergreen oak (*Quercus calliprinos* type; notably in Structure B) and some pistachio (*Pistacia* sp.) and pine (*Pinus halepensis*) (SI Appendix 5). The relatively high oak pollen in Structure B may indicate that, in some cases, the olive fruits were gathered on the mountain, or in the coastal Kurkar region, possibly from isolated stands of this tree. Wood remains of oak and some other plants typical of both Mount Carmel and the coastal plain, before the recent deforestation, identified in the structures (SI Appendix 4) may confirm this indication. Chenopodiaeae, many of which are halophytes, are present and clearly represent the saline environment of these coastal installations. Similarly, the abundant Apiaceae pollen in the submerged fossil clay paleosols off the Mount Carmel coast, mainly of the *Bunium* type, characterize these coastal saline/brackish environments. These paleosols are rather poor in olive pollen, suggesting that olive trees did not grow in the immediate vicinity of the coastal sites^55,56^ and see SI Appendix 5. It has been shown that olive pollen is mostly abundant in soil samples derived from, or very near to, olive groves^57^.

The pollen from the olive-oil extraction waste (*gefet*/*jift*) stands out. Samples from the pits at Kfar Samir^28^ are notable for their prevalence of olive pollen, between 23 and 35%. Even higher olive pollen percentages were found in recent *gefet* directly collected from an active oil mill on Mount Carmel (43%; SI Appendix 5), and are thus typical of olive-oil extraction debris. While the recent *gefet*'s pollen spectrum represents the actual environment of the gathered fruits, the pollen spectra derived from the clay paleosols around the sites have most probably undergone mixing with additional pollen over the years. The markedly low olive pollen frequencies at Hishuley Carmel may be the result of the pollen grains having been washed away, especially if these structures were used for debittering by repeated soaking in sea water.

## Discussion

### The function of the Hishuley Carmel structures

In order to reconstruct the conditions under which the Hishuley Carmel structures functioned, we examined Holocene sea-level changes and coastal migration in the northern Carmel coast based on archaeological and geomorphological markers^58^. It is estimated that sea level was ca. 4–5 m below the current level, and that the coastline stretched some 200 m to the west (i.e., ~ 180 m from the structures) (SI Appendix 8). Under humid conditions, such as those prevailing so close to the Mediterranean Sea, it is unlikely that uncured olives could be kept in closed installations for more than a few days. This, as freshly-harvested olives contain a considerable amount of water [50–70%, ^59^] and tend to suffer from fungal attacks or are spoiled when stored *en masse* without proper ventilation^60^. This also holds for dried salted olives. Yet, while immersed in salty sea water or mixed with salt, olive fruits could have been processed inside these sealed installations for a relatively lengthy period of time.

There is no similarity between the olive processing installations documented at Hishuley Carmel and Kfar Samir, such that it is highly unlikely that oil extraction was undertaken in the former installations. Indeed, the differences between the two in the architecture of the structures containing the olive pits, their associated material culture, the percentages of whole and halved olives and differences in patterns of olive pit fragmentation, implies that different kinds of olive processing took place at each of them. We suggest that while the Kfar Samir olives represent a typical crushed waste from olive-oil production^28^, the Hishuley Carmel structures were used to prepare table olives for consumption, probably by curing them in sea water or in salt. The stone-built installations would have been ideal for dry-salting since this can be done under ambient temperature. Furthermore, since the structures would not have been water-tight, liquid from the dehydrating olives could have evaporated or drained-off.

The possibility that the structures were used for storing olive oil waste, which is commonly used as fodder (e.g.,^61,62^) is unlikely, as whole olive pits are not considered waste of oil extraction (see above). Moreover, the structures were close to the Neolithic coastline and were unsuitable for long-term storage of fresh olives. Ben Salem and Znaidi^63^ already showed that special conditions are required for long-term storage of fresh olives due to their high moisture content, thus it is unlikely that the pits recovered represent stored olives.

### Wild or early cultivated trees

The radiocarbon ages, date the Hishuley Carmel structures used to prepare table olives to the first half of the 7th millennium BP, 6,700–6,500 years ago, placing them in the Middle Chalcolithic, a period that lasted some 800 years^64^. Substantial architectural remains belonging to this culture were recovered for example, at the site of Tel Tsaf located in the Jordan Valley, where stone-built dwellings, a water well and round silos were recovered, as well as hundreds of charred olive pits^65^, and olive wood^23^. The data from Hishuley Carmel, Tel Tsaf and other Chalcolithic sites, including those to the east of the Jordan River^27,32^, then corroborate the augmented exploitation of olives at this time that is also illustrated in the regional pollen record as detailed above. The question arises whether the olives were cultivated or wild by this time?

The earliest cultivated fruit trees of the Fertile Crescent were species that required the selection of superior individuals among seedlings. Afterward, the desired genotypes were vegetatively propagated, which led to uniformity among the trees^5^. Thus, variation in morphological traits of fruit and pits among trees of the same cultivar are expected to be smaller than those of naturally-growing trees of their ancestors. Indeed, following other studies, Kislev^30^ argued that the large morphological heterogeneity of olive pits found in the Kfar Samir olive-oil processing assemblage, negated their originating from cultivated trees, as compared to wild forms, as in cultivars, the range of variation in pit size reduces. Pit size per se is generally considered an acceptable, but not an absolute criterion, for the identification of olive cultivars versus the wild var. *sylvestris*^66^. However, it is sometimes controversial^67^ since for example, pits in most cultivars are significantly larger than wild types, but some modern varieties have short pits and so resemble wild populations^68,69^.

In our study, we found significant differences (ANOVA test) and metric variability (Fig. 7) amongst the olive pits from the inundated archaeological sites studied here as well as in the wild/feral populations when compared to pits of the two local cultivars (Figs. 5, 7). Indeed, previous results showed that the wild/feral trees we sampled, are genetically distinct from cultivated olives^10^. However, given the relatively late date of the Hishuley Carmel site, the distinct rise in percentages of olive pollen documented in the region at this time^22,23^, coupled with the morphometric variability found in the investigated ancient pits, it seems that the olives from the Hishuley Carmel and Kfar Samir sites were collected from wild trees. However, the possibility that they originated from early cultivated trees cannot be ruled out.

### Summary-olive exploitation on the Carmel coast

The finds from the inundated prehistoric Carmel coast sites reveal, for the first time, a well-dated three-stage-sequence for the evolution of olive exploitation in this region:

- 1. At the submerged Pre-Pottery Neolithic C site of Atlit Yam (~ 9,200–8,500 BP), and in earlier terrestrial sites in the region, olive trees were part of the natural vegetation (e.g.^55,56^) and were exploited as fire wood or as construction material, as attested by charred wood recovered (e.g.^24^). Notably, no olive pits were found at Atlit Yam despite careful sieving of *in-situ* deposits that were exceptionally rich in botanical materials with thousands of seeds, representing ~ 90 different plant species^70,71^. Given the scale, diversity and good preservation of the botanical remains at this site, it seems likely that if olive fruit was exploited on the Carmel Coast at this time, at least some traces of their remains would have been found. This leads us to conclude, that in the PPNC in this region, olive fruit was very rarely or not exploited.
- 2. In the Late Pottery-Neolithic/Early Chalcolithic site of Kfar Samir (~ 7,500–7,000 BP), thousands of crushed olive pits, associated with stone basins and woven baskets identified as strainers, were interpreted as waste from olive-oil extraction, suggesting that olive fruits were first used for olive-oil production^28^. That oil production preceded table olive consumption is logical given its important role as a food (it has a much higher caloric value than that of preserved table olives; 900 cal/100 g *versus* 138 cal/100 g respectively –^72^, and as fuel for lamps and for ceremonial purposes^73^. The discovery of olive-oil residues in a pottery vessel from the site of ‘Ain Zippori, which is contemporaneous to Kfar Samir^28,30^, further supports the early use of olive oil in northern Israel at this time.
- 3. During the subsequent Middle Chalcolithic (~ 6,600 BP), based on all the factors outlined above, we provide evidence for the production of pickled or dry-salted table olives for human consumption at the site of Hishuley Carmel. This was facilitated by easy access to sea water and/or sea salt for this process. Thus, preparation of table olives for consumption on the Carmel coast, and possibly in other regions in the Southern Levant, post-dated olive-oil production by several centuries.

The morphometry of the whole pits from Hishuley Carmel resembles that of the slightly earlier finds from Kfar Samir. Although the latter were initially identified as belonging to wild fruits^30^, the new data from Hishuley Carmel combined with the pollen evidence^22,23^, raises the possibility that some of the olives, may have belonged to early cultivated trees.

The availability of wild olive trees on the nearby slopes of Mount Carmel and on the coastal *kurkar* ridges, as attested to in the regional pollen record^55,74^, together with the close proximity of the site to the sea (i.e., availability of sea water and salt), must have played key factors in promoting the exploitation, and perhaps cultivation, of olives at Mount Carmel coastal sites such as Hishuley Carmel. The focus on olive products may, in itself, be a reflection of the early steps in olive domestication by the first half of the 7th millennium BP.

## Supplementary information

Supplementary Information.
