# Supplementary material for: Early production of table olives at a mid-7th millennium BP submerged site off the Carmel coast (Israel)
Source: Sci Rep. 2021 Jan 26;11:2218. doi: 10.1038/s41598-020-80772-6 (PMC7838305; doi:10.1038/s41598-020-80772-6)
Supplement: Supplementary file 1 — Supplementary Information. [file 41598_2020_80772_MOESM1_ESM.docx]

**Supplementary information for:
Early Production of Table Olives at a mid-7th millennium BP Submerged Site off the Carmel Coast (Israel)**

Galili E.*, Langgut D., Terral J.F., Barazani O., Dag A., Horwitz L. Kolska., Ogloblin I., Rosen B., Weinstein-Evron M., Chaim S., Kremer E., Lev-Yadun S., E. Boaretto, Ben-Barak-Zelas Z., Fishman A.

*Corresponding author, email: [galilish@netvision.net.il](mailto:galilish@netvision.net.il)

**This Supplement PDF file includes the following appendices:**

**SI Appendix 1:** Background-the Neolithic of the Southern Levant, the inundated settlements off the Carmel coast and the Hishuley Carmel and Kfar Samir sites. Authors: E. Galili, I. Ogloblin and L. Kolska Horwitz.

**SI Appendix 2:** Analysis of the building stones from Hishuley Carmel site. Author: I. Ogloblin.

**SI Appendix 3a:** Radiocarbon dating of olives from Structure A in Hishuley Carmel site. Author: Beta Analytic Radiocarbon Dating Laboratory, Miami, Florida.

**SI Appendix 3b:** Radiocarbon dating of olives from Structure B in Hishuley Carmel site in the Poznań Radiocarbon Laboratory. Author: T. Goslar.
**SI Appendix 3c:** Radiocarbon dating of olives from Hishuley Carmel and Kfar Samir sites

**SI Appendix 4:** Fragmentation patterns of Hishuley Carmel and Kfar Samir olives and wood remains recovered from the two structures in Hishuley Carmel site. Authors: D. Langgut and E. Kremer.

**SI Appendix 5:** Pollen analysis from Structures A and B at Hishuley Carmel site. Authors: M. Weinstein-Evron and S. Chaim.

**SI Appendix 6:** Experiment: The use of seawater in fermentation of olives. Authors: Z. Ben-Barak-Zelas and A. Fishman.

**SI Appendix 7:** Pilot experiment of dry salting of olives picked from naturally growing trees on the Atlit ridge. Author: E. Galili.

**SI Appendix 8:** Holocene sea-level changes and coastal changes in the Carmel coast. Author: E. Galili.

**SI Appendix 1:** **Background-the Neolithic of the Southern Levant, the inundated settlements off the Carmel coast and the Hishuley Carmel and Kfar Samir sites**

E. Galili, I. Ogloblin and L. Kolska Horwitz

**The Underwater Neolithic Sites on the Carmel Coast**

Until the 1980’s information on Levantine coastal Neolithic communities was relatively scarce and their role in the Neolithic Revolution was barely understood. This has changed following the discovery of 15 submerged coastal Neolithic settlements that are concentrated along a 17 km strip of the northern Carmel coast between Haifa and Habonim (Text Fig. 1). The sites were inundated following sea-level rise in the Holocene [1, 2]. Their archaeology has been studied sporadically sine the 1960’s, but their systematic study began in the 1980s [3-5].

The remains of the settlements are embedded in the upper part of a hard clay paleosol of terrestrial origin dated to the upper Pleistocene [6]. The duration of each site was relatively short with no earlier or later occupations as a result of continuous, post-glacial sea-level rise. Thus, the occupation phases are distributed horizontally, rather than being stratified vertically. The perfect preservation of the sites and their contents suggests that prior to inundation they were shielded by a layer of sand 1–2 m thick that prevented damage and destruction during the inundation. As a result, these submerged prehistoric villages are among the best preserved in the world. However, in recent decades, marine erosion enhanced by human activities and severe winter sea storms, has caused exposure and some destruction and erosion of parts of the sites. It has also facilitated the discovery of these substantial finds that shed light on the lifeways of these early Mediterranean coastal farming communities. The exposure of parts of the sites is accidental, and unpredictable but given the scale of the sites and budgetary limitations, excavation was limited and we primarily allowed the sea to do the job of removing the overlying sediments. Thus, after a storm, sites are surveyed by diving or snorkeling, to locate newly exposed areas and finds. The loose sand covering the finds is removed by hand-fanning, by a trowel, or by a dredging system operated with a pump. Finds are mapped and collected if endangered [for details see 7].

The submerged sites represent two main cultural phases: The Pre-Pottery Neolithic C, dated to ca. 9,250–8,000 cal. BP and represented by the site of Atlit-Yam, and the Late Pottery Neolithic (LPN) and Chalcolithic (Ch) dated to between ca. 8,000 and 6,500 cal. BP.

**Pre-Pottery Neolithic C site of Atlit-Yam:** This site is located in the north bay of Atlit, some 200-400 m offshore, at a depth of 8-12 m and covers approximately 40,000 m^2^. Radiocarbon dates for the site give a range of 9180-8550 years cal. BP for the occupation. Excavations have revealed foundations of rectangular stone structures, round installations, a structure built of sandstone megaliths, anthropomorphic stone steles, stone-built water wells and tens of hearths with charcoal remains. Seventy-five human skeletons, mostly buried in flexed positions, were uncovered in and around the structures [8]. Innumerable stone tools were recovered (axes, spearheads, sickle blades and arrowheads), as well as organic remains (animal and fish bones, numerous charred and waterlogged seeds, tree branches and pollen). The finds suggest that by the beginning of the 7^th^ millennium cal. BCE the traditional Eastern Mediterranean agro-pastoral-marine subsistence system had evolved among indigenous maritime communities, and combined agriculture and animal husbandry with hunting, foraging and marine resource exploitation [9].

**The Late Pottery Neolithic/Chalcolithic** **(LPN/Ch)** **sites:** Numerous remains from the LPN/Ch period were revealed in a narrow, almost continuous submerged belt (15 km long and 200 m wide), parallel to the present shoreline off the northern Carmel coast. The settlements recovered are: Kfar-Samir (north, center and south), Kfar-Galim (north, south), Nahal Galim, Hahotrim, Tel-Hreiz, Megadim, Neve-Yam (north, south and Temanun islet) and most recently the sites of Habonim and Hishuley Carmel [10-12]. Most of the archaeological material from these sites was collected in the course of underwater surveys, including finds washed up on the beach after storms, plus during several small scale few excavations. In these sites, stone and wood-built structures were found including water wells constructed of alternating layers of wooden branches and stones. Other finds from the LPN/Ch sites comprise pottery, groundstone, bone and flint artefacts, ornamental figurines, large assemblages of animal and botanical remains and stone-built cist graves containing human skeletons. The subsistence of these settlements was characterized by an increased reliance on farming and animal husbandry and use of secondary animal products (milk products, wool fibres, etc.) in addition to the exploitation of marine resources and hunting.

**The Kfar Samir site:** The LPN/Ch site of Kfar Samir is located 1–200 m offshore and at a water depth of 0–5.5 m. The remains form a scatter that runs parallel to the coastline for ~1200 m. Due to its size, the site was randomly divided into three sectors: north, central, south. The oil-extraction complex (pit 3 described in the main text), was found in the central sector, together with areas of paving made of unworked slabs and stones. No clear domestic structures were recognized, but several hearths were found containing burnt bones and charcoal. On the north-western part of the central sector and ~200 m offshore three water wells built of stone and wood were found at a depth of 5.5 m. Their fill contained fragments of stone and clay, flint artefacts, animal bones, olive pits and other plant remains. A well-preserved bowl made of carob-wood was found on a section of stone paving [For details see 13].

**The Hishuley Carmel site:** The Hishuley Carmel site (32º 46' 37.69'' N; 34º 57' 13.36'' E, text Fig. 1b) is located on the 10 km long coastal strip stretching between Haifa in the north and Atlit in the south [10]. The site is ca. 1 km south of the southern sector of Kfar Samir and 1800 m south of the oil extraction complex (Installation 6 described in the main text). The Hishuley Carmel site was regarded until recently as the southern extent of Kfar Samir site. In 2018 it was given a separate name due to the unique and freshly-exposed finds described here, which suggest that it is slightly later than Kfar Samir site and that it may have functioned as an independent manufacturing zone.

The south–north oriented coast in this area is straight, sandy and exposed to the waves of the open sea. The sea bottom is flat, and slopes slightly westward. The site is located at 0-100 m from the shore, and the prehistoric finds occur at depths of 0-4 m below sea level. The prehistoric remains associated with Kfar Samir are embedded in the uppermost layer of the clay paleosol, while finds associated with shipwrecks from historical periods (mainly iron and stone anchors) are scattered on the surrounding sea bottom. The archaeological horizon is usually covered by sand whose thickness varies depending on the season and associated waves and sea currents. Aside from the two structures containing the olive pits that are described in the article, four round installations (up to 1.5 m in diameter) made of undressed stones were identified ~80m offshore at 3m depth (Fig. 1).


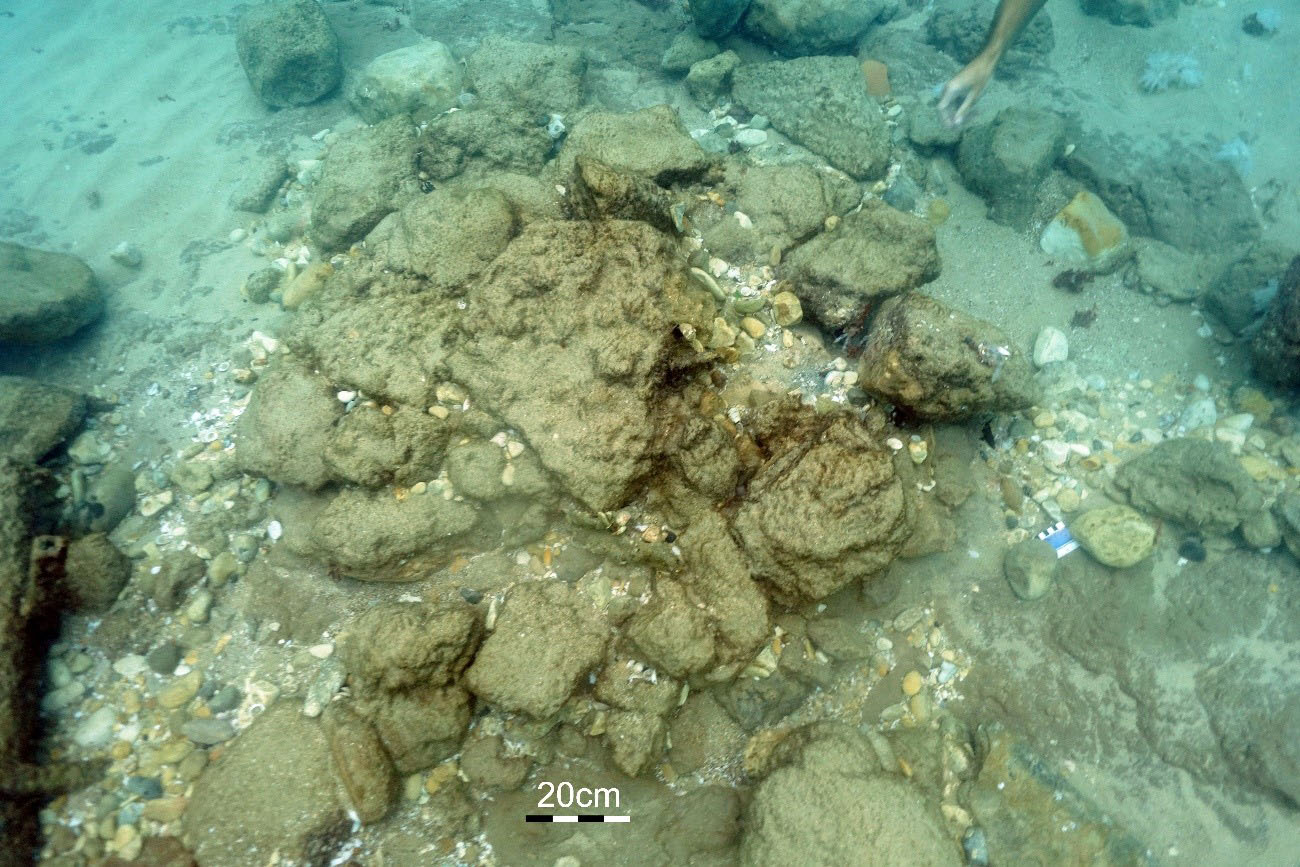


Fig. 1. One of the round structures made of undressed stones from Hishuley Carmel (Photo E. Galili)

**Methods used in Hishuley Carmel site:**

In Hishuley Carmel, no excavations were carried out. Samples of olives and sediments were collected following short episodes of exposure of the site after storms. To determine the thickness of the subsurface fill of the structures and the level of the paving layer inside the structures without disturbing their fill, an iron post (10 mm thick, 100 cm long) was hammered inside and outside of each structure. Core samples of undisturbed sediments around the structures and in their fill, were taken for pollen analysis using 40 cm long and 40 mm thick PVC pipes that were hammered perpendicularly into the soil (Figs. 2-4). The olive pits were removed from the fill of Structures A and B using a trowel and were kept in polyethylene bags filled with sea water, closed tightly and refrigerated at 4^o^C. No sieving or sorting of any kind was performed at this stage.

**The significance and importance of the submerged sites and their preservation**

The submerged Neolithic sites on the Carmel coast of Israel are of critical importance for prehistoric research in the Levant since they provide information on the lifestyle of past communities in a unique and previously understudied habitat, the Levantine littoral. Notably, it is of interest to study how people adapted to the special conditions on the coast - sandy soils, marshy areas, marine resources, sea storms and hurricanes, limited fresh water etc. These adaptations are manifest in the archaeological record in special architectural features (such as the digging of water wells, well-spaced village layout, construction of a seawall to stop the rising sea level), a distinctive, broad-based subsistence economy (fishing-farming and animal husbandry) and specialized technologies (sinkers, fish hooks, wood working tools such as adzes perhaps for making boats, lithic daggers for killing/cleaning fish), amongst many other features.

In addition, the excellent preservation of archaeological finds in these sites far exceeds that of contemporaneous terrestrial ones. Consequently, the corpus of finds, that includes wooden and woven objects, rich assemblages of botanical remains etc., offers valuable insights into aspects of Neolithic life which are missing from their terrestrial counterparts and so complete our knowledge of the Neolithic period. Moreover, the submerged sites are the only ones in the world containing all elements that make up an entire village, including: domestic dwellings, water wells, different types of installations (e.g. rubbish pits, industrial features), enclosures, symbolic structures and human burials. Finally, these villages also provide invaluable information on Holocene sea-level changes in the Eastern Mediterranean, and human responses to them.

In recent years, erosion due to sea level rise and human activity is destroying these sites, necessitating constant survey and focused excavation where needed. To preserve certain structures, sand-filled bags have been placed to bolster them, and after a decade these appear to be intact and still doing their job. Research and publication of
finds and other aspects relating to these sites are ongoing.


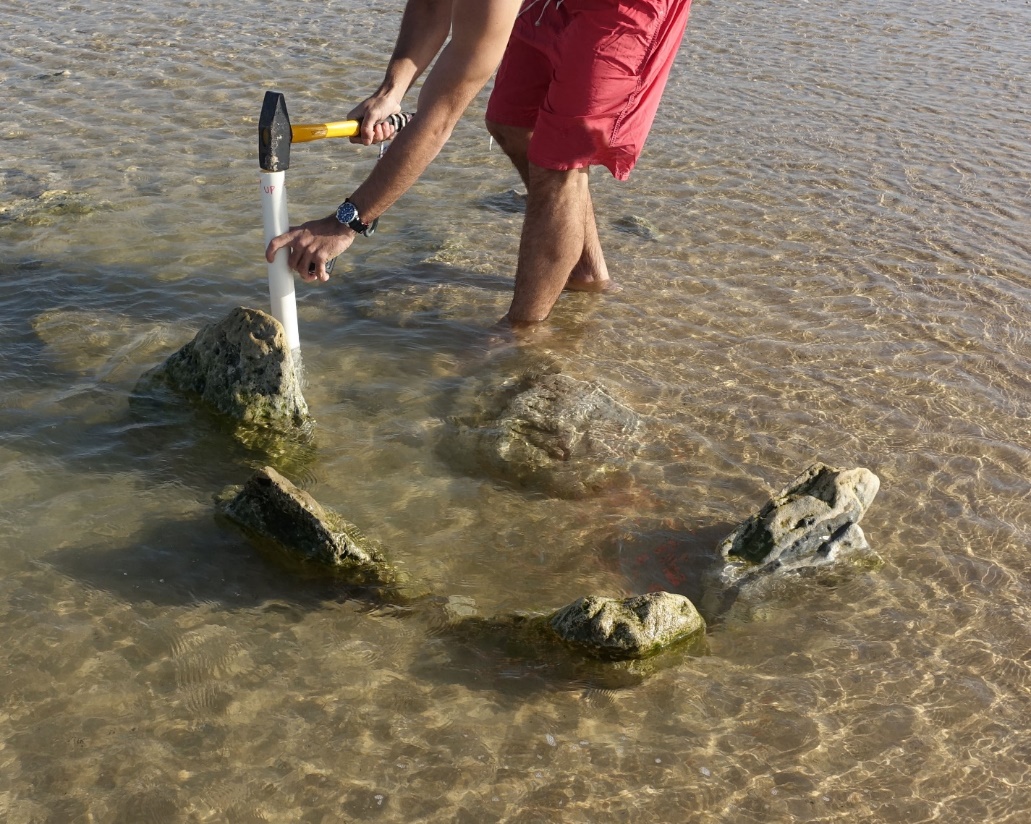


Fig. 2. Core sampling for pollen in structure A during low tide (Photo E. Galili)


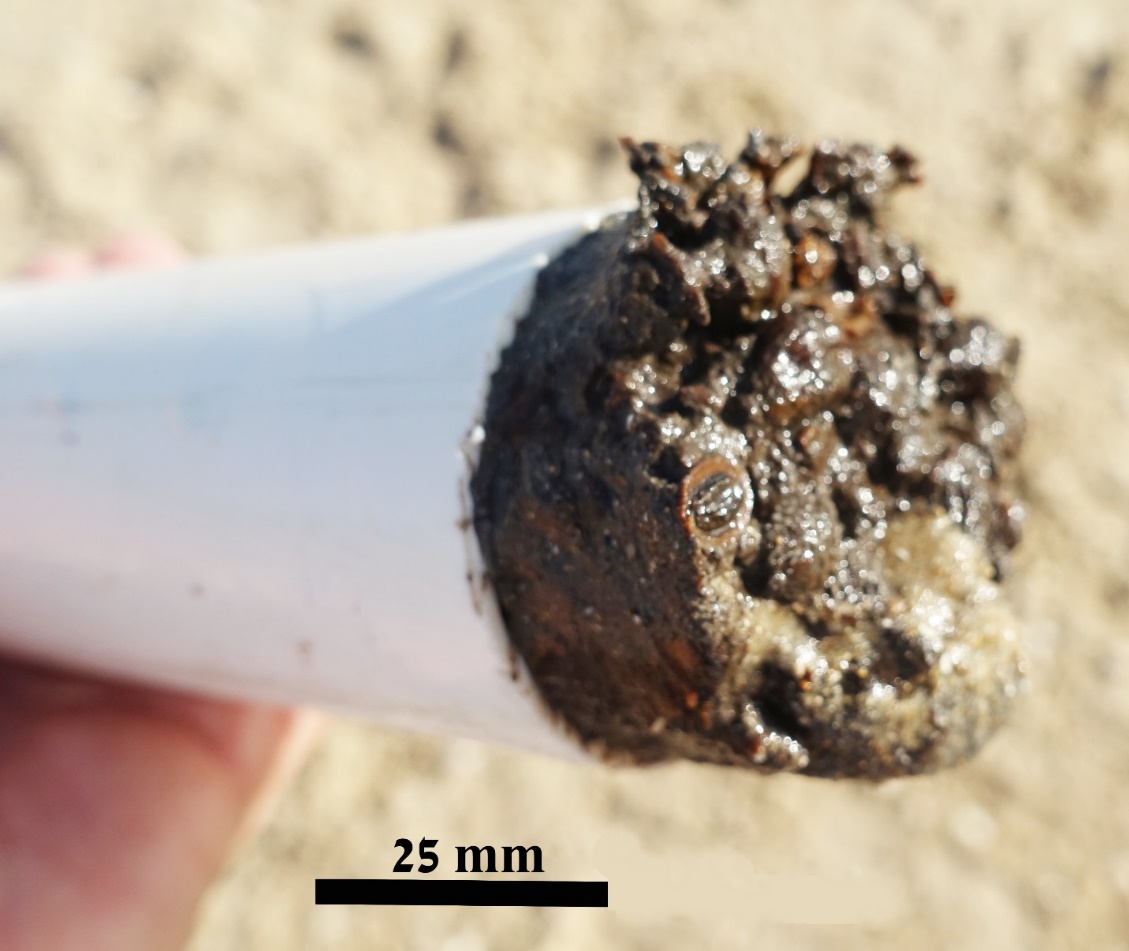

Fig. 3. The PVC tube containing the core sample, with traces of olive pits (Photo E. Galili).


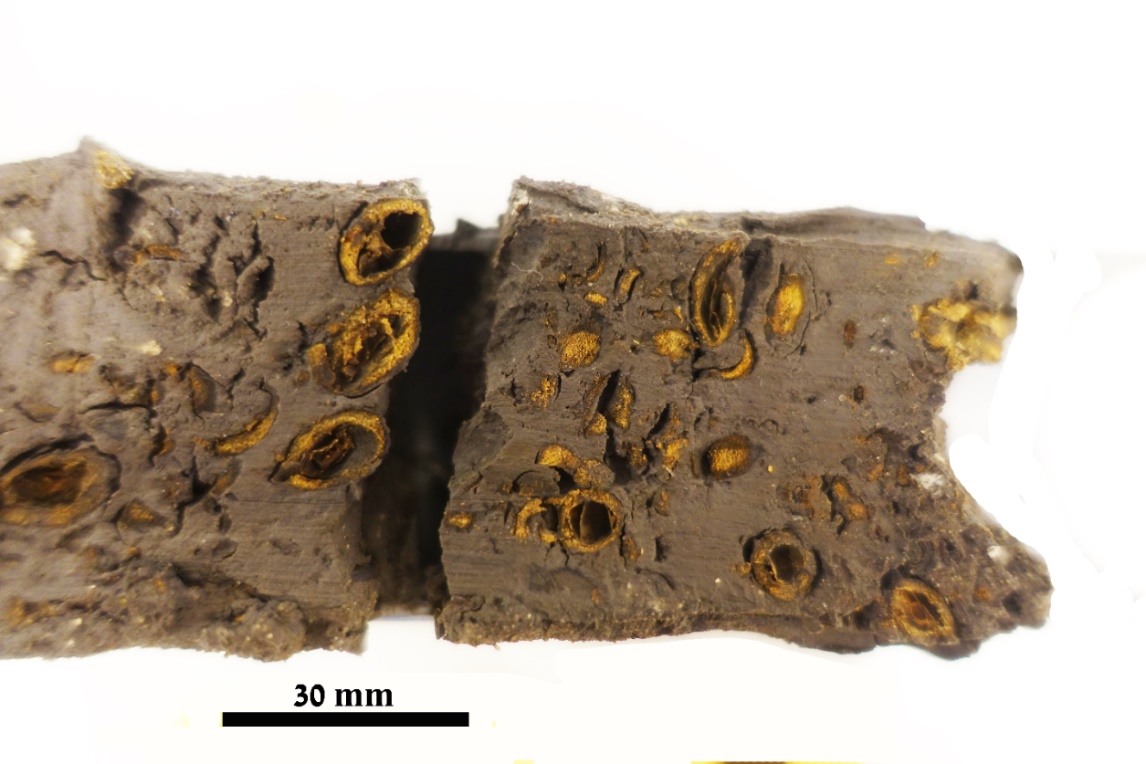


Fig. 4. Cross-section in the core sample taken in structure A (Photo E. Galili).

**References**

1. E. Galili E., M. Weinstein-Evron M. and A. Ronen, Holocene Sea-Level Changes Based on Submerged Archaeological sites Off the Northern Carmel Coast in Israel. *Quaternary Research* 29: 36-42 (1988).

2. E. Galili and Y. Nir, The Submerged Pre-Pottery Neolithic water well of Atlit-Yam, northern Israel, and its palaeoenvironmental implications. *The Holocene* 3: 265-270 (1993).

3. E. Galili, M. Weinstein-Evron, Prehistory and paleoenvironments of submerged sites along the Carmel Coast of Israel. *Paléorient* **11** (1), 37-52 (1985).

4. E. Galili *et al.,* Atlit-Yam: A Prehistoric Site on the Sea Floor off the Israeli Coast. *Journal of Field Archaeology* 20: 133-156 (1993).

5. E. Galili, Ph.D. Dissertation, Tel Aviv University, Faculty of Humanities, Department of Archaeology and Near- Eastern cultures, *Submerged Settlements of the Nin^th^ -seven^th^ mill. BP off the Carmel Coast* (Hebrew)
(2004).

6. Y. Sneh, M. Klein, Holocene sea level changes at the coast of Dor, southeast Mediterranean. *Science* **226**, 831-832 (1984).

7. E. Galili, L. Kolska Horwitz and B. Rosen, The Israeli model for the detection, excavation and research of submerged prehistory. TINA Maritime Archaeological Periodical, *Periodical Publications of the Turkish Archaeological Foundations*: Pp. 31-69 (2019).

8. E. Galili, V. Eshed, A. Gopher and I. Hershkovitz, Burial practices at the submerged PPNC site of Atlit- Yam, Northern coast of Israel: What do they tell us about the final phase of the Pre- Pottery Neolithic culture*. BASOR* 339: 1-19 (2005).

9. E. Galili, B. Rosen, A. Gopher and L. Kolska Horwitz, The Emergence and Dispersion of the Eastern Mediterranean Fishing Village: Evidence from Submerged Neolithic Settlements off the Carmel Coast, Israel. *Journal of Mediterranean Archaeology* 15.2: 167-198 (2002).

10. E. Galili, B. Rosen, Atlit Yam- preliminary report. *Hadashot Archaeologiot* **125**. <http://www.hadashot-esi.org.il> (2013).

11. E. Galili, L.K. Horwitz, V. Eshed, B. Rosen, “Submerged Pottery Neolithic settlements off the coast of Israel: Subsistence, material culture and the development of separate burial grounds” in Under the Sea: Archaeology and Palaeolandscapes of the Continental Shelf*,* G. Bailey, J. Harff, D. Sakellariou Eds. pp. 105-130 (Springer, 2017).

12. E. Galili, B. Rosen, M. Weinstein-Evron, I. Hershkovitz, V. Eshed, L.K. Horwitz, Israel: Submerged Prehistoric Sites and Settlements on the Mediterranean Coastline — the Current State of the Art, in: Bailey, G. *The Archaeology of Europe’s Drowned Landscapes*. Springer Nature: 443-481 (2000).

13. E. Galili, D. Cvikel, J. Benjamin *et al.,* Coastal paleoenvironments and prehistory of the submerged Pottery Neolithic settlement of Kfar Samir, Israel. *Paléorient* **44**(2), 113-132 (2018).

**SI Appendix 2: Analysis of the building stones from Hishuley Carmel site**

I. Ogloblin

The stone samples taken from the structures were analyzed using FTIR (Fourier Transform Infrared Spectroscopy) following the potassium bromide method, and thin petrographic sections (Figs. 1, 2). Using a chisel and a hammer, samples were taken from the upright stones of Structures A and B, and from the stone paving found beneath the layers containing olive remains, in order to identify their mineralogy and hence their provenance. These samples were kept in closed and marked polyethylene bags. Spectra wavenumbers were collected with a thermo scientific spectrometer using. Interpretation of the spectra was based on published studies, reference libraries [1] and field controls of stone samples collected in the region. Additionally, thin petrographic sections were made and were observed by using a polarized light microscope equipped with a DS Camera Control. The thin sections were compared to the reference collection of the Laboratory for Sedimentary Archaeology, at the University of Haifa.


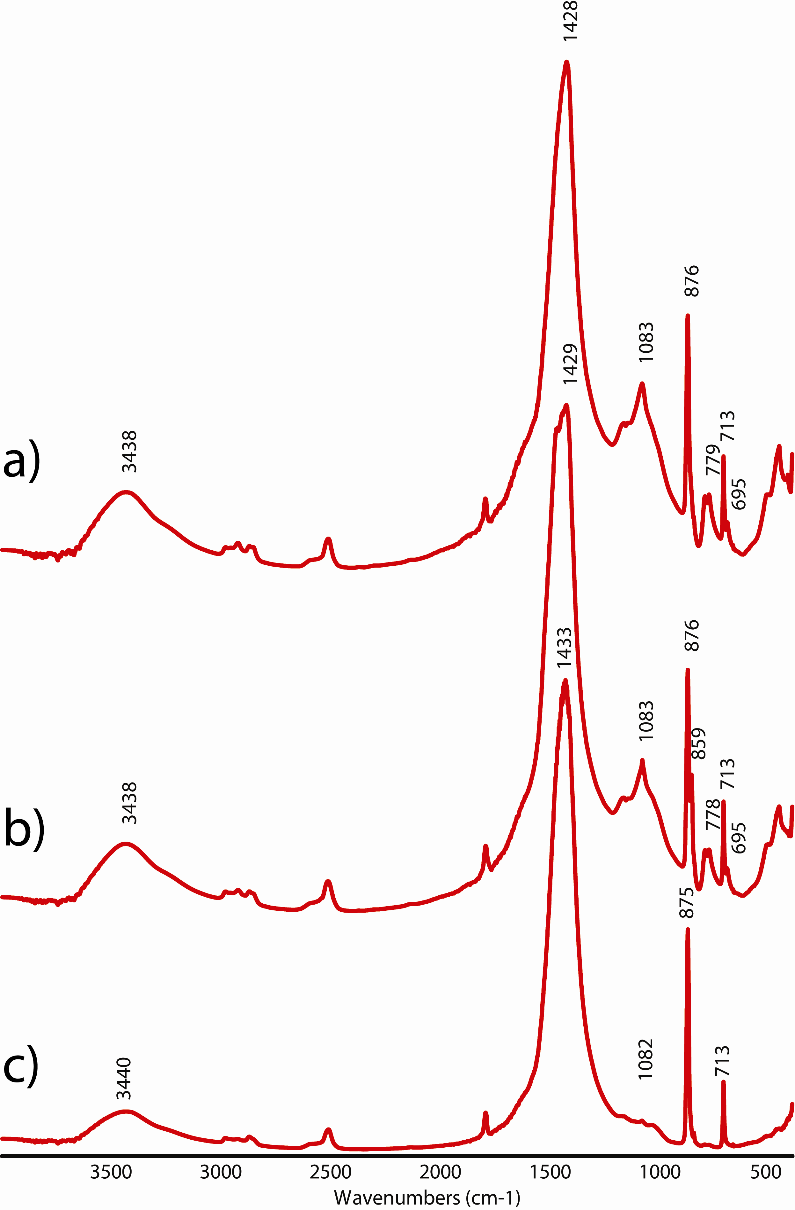


Fig. 1. Representative FTIR Spectra of stones used to construct Installations A and B. Calcite is identified based on absorption bands at 1432, 876, and 713 cm^-1^; aragonite has an extra peak at 860 cm^-1^; and quartz is based on 1083, 797, 778, and 695 cm^-1^. a) FTIR spectrum of Kurkar identified by the peaks for calcite and quartz. b) FTIR spectrum of beachrock consisting of calcite, aragonite, and quartz. c) FTIR spectrum of limestone composed of geogenic calcite [2].


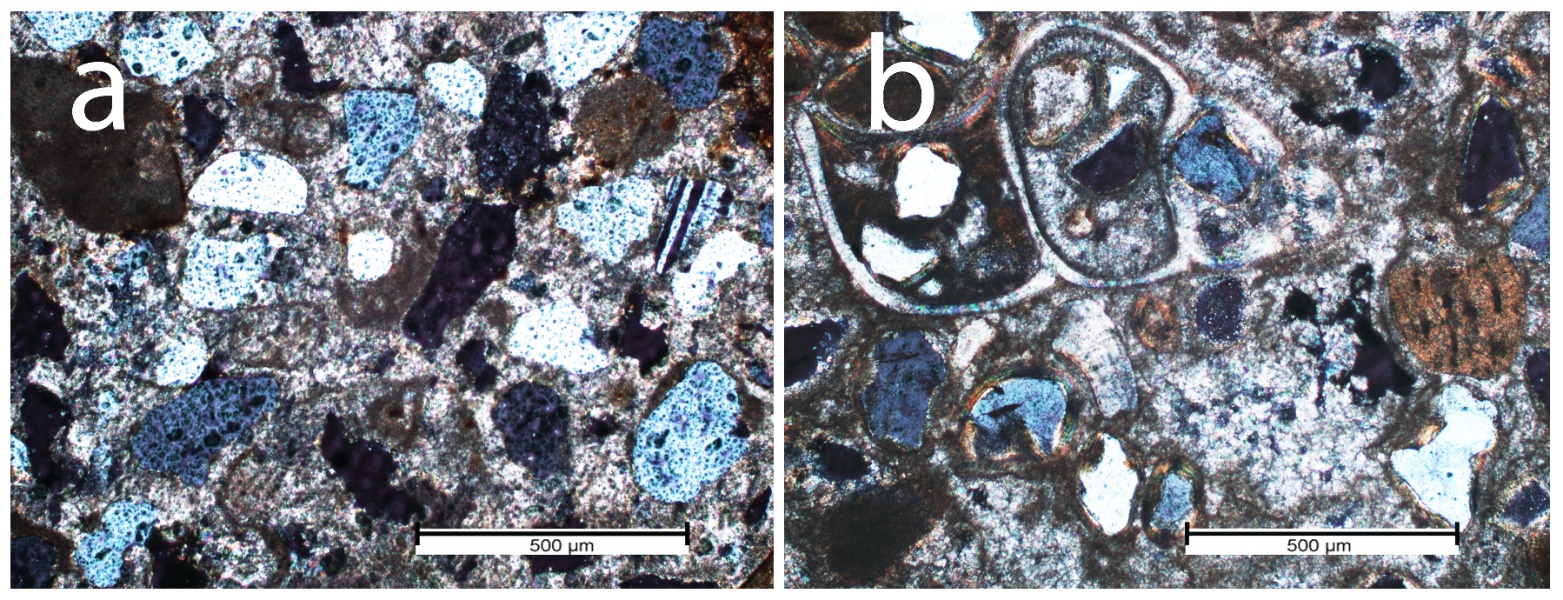


Fig. 2. Thin sections photographed under cross-polarized light from the Hishuley Carmel structures: a-Kurkar: quartz grains, feldspar and carbonate particles, cemented by sparitic calcite. b Beachrock: whole and broken marine shells and quartz grains, cemented by aragonite (Photo I. Ogloblin).

**References**

1. 73. S. Weiner, Microarchaeology: Beyond the Visible Archaeological Record. (Cambridge University Press, 2010).

2. L. Regev, K.M. Poduska, L. Addadi, S. Weiner, E. Boaretto, distinguishing between calcites formed by different mechanisms using infrared spectrometry: archaeological applications. *J Archaeol Sci* **37**, 3022-3029 (2010).

**SI Appendix 3a: Radiocarbon dating of olives from Structure A in Hishuley Carmel site**

Beta Analytic Radiocarbon Dating Laboratory, Miami, Florida

**Sample preparation**

Each sample was first visually inspected for size and durability.  Each olive pit sample was split open and the pulp was removed and discarded.  The inner parts of the outer “shell” were then reduced to small particles by gently crushing and they were then saturated in de-ionized water at 70C.  Each was then soaked in 1N HCl for 1-2 hours and repeated, if needed, to eliminate any carbonates present (none were noted).  The sample was then rinsed to neutral with deionized H2O.  A 1-2% alkali solution was then applied (50/50 wt/wt% NaOH) at 70C and the sample was allowed to soak for 2-4 hrs. The sample was rinsed multiple times with deionized H2O and the alkali applications and rinsing repeated until no color change was observed on the application of fresh NaOH.  It was then rinsed to neutral with deionized H2O.  A final hot acid wash (0.5-1.0 N HCl) was applied to ensure the alkali was neutralized and once again rinsed to neutral with deionized water.   During this process, any roots and organic debris were eliminated.  The sample was vacuum desiccated for 12-24 hours.  It was then microscopically examined for cleanliness, uniformity and where applicable appropriately sub-sampled for the d13C and AMS measurements.

The two samples from Hishuley Carmel Structure A gave the radiocarbon ages:

**Laboratory number Beta-516499**: Conventional radiocarbon age 5,750 ± 30 BP.

**Laboratory number Beta-516500:** Conventional radiocarbon age 5,780 ± 30 BP 95.4%.

**SI Appendix 3b: Radiocarbon dating of olives from Structure B in Hishuley Carmel site in the Poznań Radiocarbon Laboratory (order 14203/18)**

T. Goslar

**Procedure of 14C dating with the AMS technique, consists of a few stages:**

**a) Chemical pre-treatment:** Methods of chemical pre-treatment generally follow those used in the Oxford Radiocarbon Accelerator Unit, as described by Brock *et al.* [1]. Samples of charcoal, wood, peat, or other plant remains and of wool (after mechanical removal of macroscopic contamination visible under binocular) are treated with 0.25M (UV1) - 1M (UV, UW, WW, ZR, NN) HCl (80°C, 20+ min), 0.025-0.1M NaOH (room temperature for fragile plant remains or wool (UV, UV1, NN), or 80°C for wood, peat and charcoal (UW, WW, ZR) and then 0.25M HCl (80°C, 1 hour). After treatment with each reagent, the samples are rinsed with deionised water (Millipore) until pH=7. For the first HCl treatment, longer time (20+) is applied if emanation of gas bubbles from sample is still visible. The step of NaOH treatment is repeated a few times, generally until no more coloration of the NaOH solution appears (coloration of solution is caused by humic acids dissolved in NaOH).

**b) Production of CO_2_ and graphitisation:** In the case of organic samples, CO_2_ is produced by combusting the sample. Combustion of organic samples is performed in closed (sealed under vacuum) quartz tubes, together with CuO and Ag wool, in 900°C, over 10 hours. The obtained gas (CO_2_ + water vapour) is then dried in a vacuum line, and reduced with hydrogen (H_2_), using 2 mg of Fe powder as a catalyst. From June 2017 on, combustion of bigger samples of grains, charcoal, wood and collagen, and reduction of the obtained CO_2_ has been performed with an automated graphitisation system AGE [2] coupled with elemental analyser vario ISOTOPE select. The obtained mixture of carbon and iron is then pressed into special aluminium holder, according to the description provided by Czernik and Goslar [3]. In the same way standard samples are prepared, i.e. samples not containing ^14^C (coal or IAEA C1 Carrara Marble) and samples of the international modern ^14^C standard (Oxalic Acid II).

**c) AMS ^14^C measurement:** Measurements described were performed in the AMS ^14^C Laboratory of the A. Mickiewicz University in Poznań. Cooperation between the Poznań Radiocarbon Laboratory and the AMS ^14^C Laboratory is regulated by the Agreement between Foundation of the A. Mickiewicz University and the A. Mickiewicz University. Content of ^14^C in a sample of carbon is measured using the spectrometer “Compact Carbon AMS” described in the paper of Goslar *et al.* [4]. The measurement is performed by comparing intensities of ionic beams of ^14^C, ^13^C and ^12^C measured for each sample and for standard samples (modern standard: “Oxalic Acid II” and standard of 14C-free carbon: “background”). In each AMS run, 30-33 samples of unknown age are measured, alternated with measurements of 3-4 samples of modern standard and 1-2 samples of background. In case, where organic samples are dated, the background is represented by coal, while in case of carbonate samples, the background is represented by the sample IAEA C1.

**d) Calculation of ^14^C age and calibration of ^14^C age:** Conventional ^14^C age is calculated using a correction for isotopic fractionation according to Stuiver and Polach [5], based on a ratio ^13^C/^12^C measured in the AMS spectrometer simultaneously with the ratio ^14^C/^12^C (note: the measured values of δ ^13^C depend on isotopic fractionation during CO_2_ reduction and isotopic fractionation inside the AMS spectrometer, and as such, they cannot be compared with values of δ ^13^C determined with conventional mass spectrometers on gas samples). Uncertainty of calculated ^14^C age is determined using uncertainty implied from counting statistics, and also spread (standard deviation) of partial ^14^C/^12^C results, whichever is bigger. Uncertainties of ^14^C/^12^C ratios measured on standard samples are additionally taken into account. The 1-sigma uncertainty of conventional ^14^C age given in our reports, is the best estimate of the total uncertainty of measurement.

The two samples from Hishuley Carmel Structure B gave the following radiocarbon ages:

CAR-PIT1-A Poz-107214 5,740 ± 35 BP

CAR-PIT1-B Poz-107215 5,805 ± 35 BP

**References**

1. F. Brock, T. Higham, P. Ditchfield, C. Bronk Ramsey, Current pre-treatment methods for AMS radiocarbon dating at the Oxford Radiocarbon Accelerator Unit (ORAU). *Radiocarbon* 52 (1): 103-112 (2010).

2. L. Wacker, M. Nemec, J. Bourquin, A revolutionary graphitisation system: Fully automated, compact and simple. *Nuclear Instruments and Methods in Physics Research* B 268 (7-8), 931–934 (2010).

3. J. Czernik, T. Goslar, Preparation of graphite targets in the Gliwice Radiocarbon Laboratory for AMS 14C dating. *Radiocarbon* 43 (1): 283-291 (2001).

4. T. Goslar, J. Czernik, E. Goslar, Low-energy 14C AMS in Poznan radiocarbon Laboratory, Poland. *Nuclear Instruments and Methods in Physics Research* *B* 223-224: 5-11.
5. [Stuiver M., Polach H.A. 1977. Discussion: reporting of ^14^C data. Radiocarbon 19 (3): 355-363 (2004).](javascript:go_ref('stuiver1977dr','Stuiver',1977,'Discussion+reporting','article'))

**SI Appendix 3c: Radiocarbon dating of olives from Hishuley Carmel
and Kfar Samir sites**

E. Boaretto

All the radiocarbon dates obtained from Hishuley Carmel and Kfar Samir olive pits, that are discussed in this paper, are summarized in Table 1. The table includes seven dates from Kfar Samir Locus 6, that have already been published [1], and four dates from Hishuley Carmel (this work). All dates have been calibrated using the new calibration curve of Reimer *et al.* [2] and the probability distribution of the calibrated ranges are calculated and plotted using OxCal v4.4.2 (2020).

**Table 1:** Radiocarbon ^14^C results of olive pits from Hishuley Carmel together with the radiocarbon dates obtained from the installation of olive-oil production from Kfar Samir [1] are in the Table 1:

| **Sample location** | **Lab. No.** | **^14^C yr (BP) ±1σ** | **Calibrated age range BP**  **(68.2% probability)** | **Calibrated age range BP (95% probability)** |
| --- | --- | --- | --- | --- |
| Kfar Samir  Installation 6 | Beta 82845 | 6,080±70 | 7152 (6.6%) 7129  7011 (57.6%) 6847  6815 (4.1%) 6800 | 7160 (94.6%) 6784  6761 (0.9%) 6752 |
|  | Beta 82846 | 6,210±150 | 7271 (66.1%) 6936  6919 (2.2%) 6903 | 7424 (94.6%) 6780  6765 (0.8%) 6749 |
|  | Beta 82847 | 6,210±80 | 7248 (12.3%) 7206  7171 (7.6%) 7147  7139 (48.4%) 6997 | 7281 (95.4%) 6893 |
|  | Beta 82848 | 6,230±80 | 7253 (19.4%) 7197  7176 (8.1%) 7152  7131 (40.8%) 7009 | 7319 (95.0%) 6937  6915 (0.4%) 6906 |
|  | Beta 82715 | 6,500±70 | 7476 (14.0%) 7442  7434 (54.3%) 7323 | 7563 (4.5%) 7539  7513 (91.0%) 7273 |
|  | RT 1898 | 5,790±55 | 6660 (62.1%) 6536  6517 (6.1%) 6502 | 6734 (6.1%) 6690  6683 (89.3%) 6450 |
|  | RT 1930 | 5870±70 | 6786 (64.0%) 6624  6583 (4.3%) 6567 | 6879 (0.3%) 6874  6854 (95.1%) 6495 |
| Hishuley Carmel |  |  |  |  |
| Structure A | Beta-516499 | 5,750±30 | 6618 (1.4%) 6615  6607 (13.6%) 6585  6565 (53.3%) 6494 | 6640 (95.4%) 6451 |
|  | CAR-PIT1-A  Poz-107214 | 5,740±35 | 6621 (15.9%) 6585  6565 (52.4%) 6488 | 6640 (94.8%) 6442  6418 (0.6%) 6413 |
| Structure B | Beta-516500 | 5,780±30 | 6638 (63.5%) 6547  6513 (4.8%) 6504 | 6660 (95.4%) 6495 |
|  | CAR-PIT1-B  Poz-107215 | 5,805±35 | 6663 (68.3%) 6560 | 6726 (2.6%) 6707  6675 (92.8%) 6495 |

Fig 1: Probability distribution of the radiocarbon dates from Kfar Samir (black) and Hishuley Carmel (red and blue)


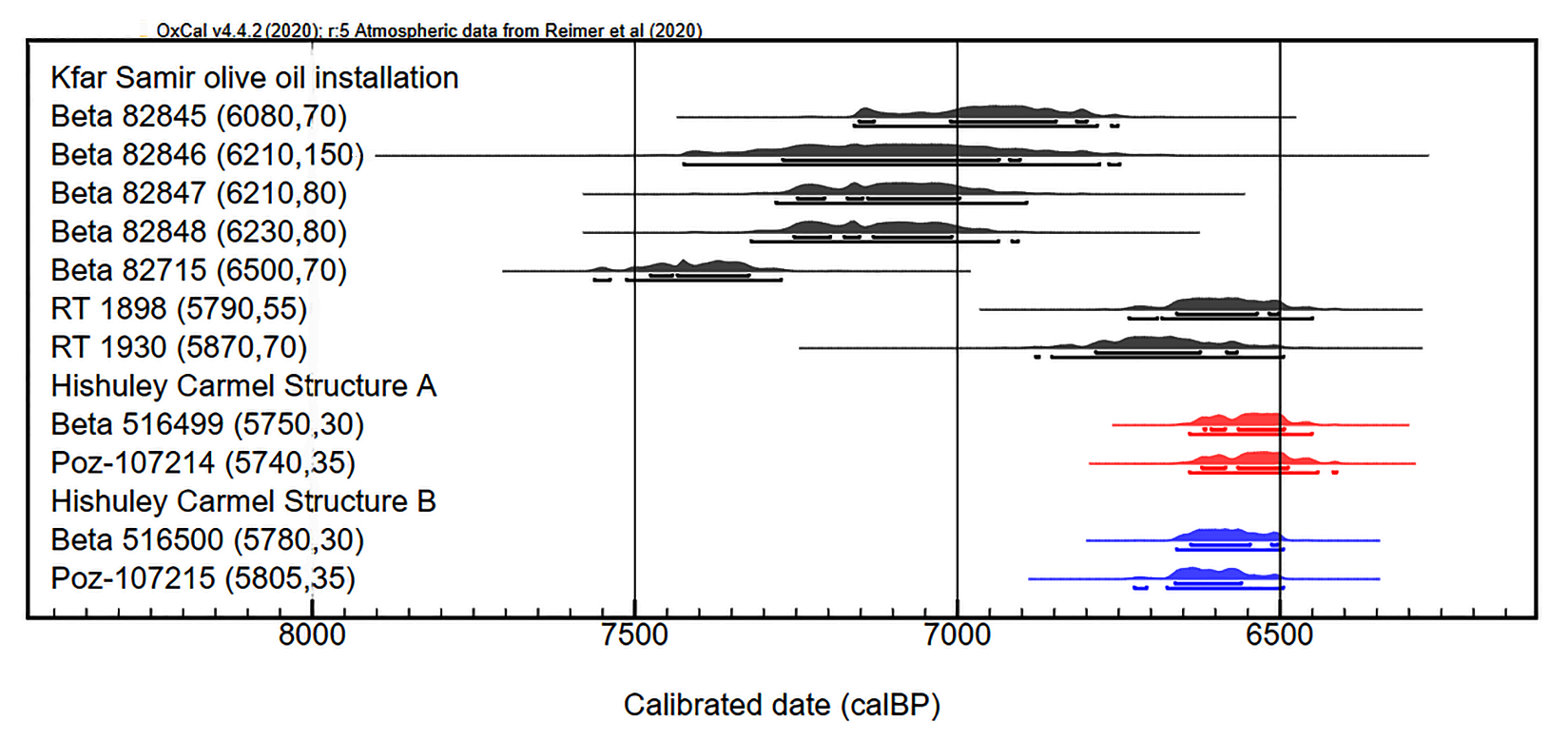


**References**

1. E. Galili, B. Rosen, M. Weinstein-Evron, I. Hershkovitz, V. Eshed, L.K. Horwitz, Israel: Submerged Prehistoric Sites and Settlements on the Mediterranean Coastline — the Current State of the Art, in: Bailey, G. *The Archaeology of Europe’s Drowned Landscapes*. Springer Nature: 443-481 (2000).

2. P. Reimer, W. Austin, The IntCal20 Northern Hemisphere radiocarbon age calibration curve (0–55 cal kBP). *Radiocarbon*, 62. (2020).

**SI Appendix 4: Fragmentation patterns of Hishuley Carmel and Kfar Samir olives and wood remains recovered from the two structures in the Hishuley Carmel site**

D. Langgut and E. Kremer

**a: Fragmentation patterns of Hishuley Carmel and Kfar Samir olives**

To assess the preparation techniques used for oil production *versus* production of table olives, two olive-pit assemblages were compared, one from Hishuley Carmel, and the other from Kfar Samir's central sector, located 1,800 m to the north (Text Fig. 1c). The same analysis was conducted on both assemblages: ancient olive pit samples were taken out of the liquid in which they were stored; the pits were then gently washed and cleaned using sea water. Later, the olive pit remains were manually sorted and separated into their relevant categories: whole pits, halved pits (broken along the longitudinal suture line) and fragmented pits (Text Fig. 5). The group of halved pits represents mostly natural breakage that occurs with time. Therefore, this category was combined with the group of the whole pits and is termed in this study ‘intact pits’. Each fraction was wrapped for two minutes in absorbent paper to eliminate excess free external water. The sorted pit fractions were weighed and counted. Since the whole pits contain an inner closed space (endosperm cavity) filled with water, the weight of the water was estimated (based on average cavity size) and subtracted to get the net weight of the whole, water-free pit. The net weight was calculated to compare it to the fragmented and halved pits that did not contain water.

**b: Wood remains recovered from the two structures in the Hishuley Carmel site**

Apart from olives, the Hishuley Carmel assemblages yielded additional botanical remains. Due to their scarcity in terrestrial sites of this period, those that were identified are listed here. They were identified using a Zeiss Stereo Discovery V2 microscope. Taxa identified at the Hishuley Carmel site were cupule and acorn remains of *Quercus* sp. (oak), a spike of *Zizyphus* *spina-christi* (jujube), a small chunk of *Phoenix* *dactylifera* (date palm) trunk, and a few indeterminable weeds primarily leaf parts. In the Kfar Samir assemblage, wood remains of *Laurus nobilis* (laurel) and *Phoenix* *dactylifera* were identified.

**SI Appendix 5: Pollen analysis from Structures A and B at Hishuley Carmel site**

M. Weinstein-Evron and S. Chaim

The samples of undisturbed sediment were extracted from the PVC tubes and were kept in marked polyethylene bags, at 4ºC for analysis. Twenty grams of sediment were processed for each sample using standard palynological techniques [1] and identified using the comparative reference collection of the Laboratory of Palynology at the Zinman Institute of Archaeology, University of Haifa, and relevant atlases and reports [e.g., 2].

The samples studied contained well-preserved pollen. Results of the pollen analysis from the structures were compared to (i) the pollen assemblages from the nearby Kfar Samir site that represent olive-oil extraction waste, (ii) pollen from modern olive-oil extraction waste, and (iii) pollen assemblages of fossil clay paleosols from the Kfar Samir site (Table 1).

The pollen samples from Structures A and B yielded extremely low olive pollen counts. Otherwise, they show inter-sample variability. For example, arboreal pollen differs between them in both ratios and composition. This may be due to the olives having been picked at different locations. Yet, both assemblages indicate a typical Mediterranean vegetation as found today in the Mount Carmel region, with mainly evergreen oak (*Quercus callipr*i*nos* type; notably in Structure B) and some pistachio (*Pistacia* sp.) and pine (*Pinus halepensis*). Other arboreal pollen types, including deciduous oak (*Q*. *ithaburensis* type), Rosaceae (*Amygdalus* type), Tamarisk (*Tamarix*) and Buckthorn (*Rhamnus*), appear in either samples.

The herbaceous pollen also differs between the samples, with varying emphases on the main components: Chenopodiaceae (goosefoot family; *Atriplex* type), Apiaceae (parsley family; mainly in Structure A), Asteraceae Asteroidae (*Aster* subfamily; mostly *Inula* type) and Poaceae (grasses). Other taxa, mainly in Structure B, include Fabaceae (legume family), Lamiaceae (mints), Malvaceae (mallows), Dipsacaceae (teasel family), Ranunculaceae (buttercups), Brassicaceae (mustards) and Rubiaceae (madder family).

Chenopodiaeae clearly represent the saline environment of these coastal installations. Similarly, the abundant Apiaceae pollen in both recent and submerged fossil clays off the Mount Carmel coast, mainly of the *Bunium* type, were shown to characterize saline/brackish environments of the coastal plain [3]. Hence, together with Chenopodiaceae, Apiaceae can be taken to reliably represent the coastal environment of the site, albeit of a somewhat less saline nature. While we cannot detail the specific plants responsible for the *Bunium*-type pollen, various Apiaceae are known spice plants. *Crithmum maritimum*, from which the *Crithmum*-type pollen was most probably derived, is quite widespread along the rocky shores of the Mediterranean. It is noted for its high Vitamin-C content [4], for which its leaves had been widely collected in antiquity [e.g., 5]. *Atriplex*, as well as other representatives of the Chenopodiaceae family, are also edible; the same holds good for many Brassicaceae. The relatively high ratios of insect-pollinated taxa (e.g., Fabaceae and Lamiaceae) may indicate some human collection. Dipsacaceae and Malvaceae pollen may indicate the presence of disturbed, ruderal environments near the site, as may also be indicated by some of the Asteracea, Apiaceae and maybe other plant families.

Pollen spectra of the nearby fossil clay paleosols from the Kfar Samir site are rather poor in olive pollen, suggesting that in antiquity, olive trees did not grow close to the two currently submerged sites [3, 6 – 9]. It has been shown that olive pollen is most abundant in soil samples derived from, or very near to, current olive groves (Text ref. 50). Pollen spectra from the sediments derived from the olive-oil extraction waste (in Hebrew *gefet*) at Kfar Samir, stand out [7] (Table 1). These pollen spectra are noted for their abundant frequencies of olive pollen, between 23% and 35%. An even higher olive pollen percentage was found in recent *gefet* directly collected from an active oil mill on Mount Carmel (43%; table 1), and is thus, typical of olive-oil extraction debris. While the recent *gefet* spectrum represents the actual environment of the gathered fruits, the pollen spectra derived from the clay palaeosols at the sites have most probably undergone mixing with additional pollen sedimented throughout the years. Still, olive pollen ratios in the *gefet* are excessively higher than in any of the other samples, retaining its indicative value for olive-oil extracted debris. As argued before [7], the high olive pollen in the *gefet* samples does not indicate that olive trees grew in the close vicinity of the Kfar Samir and Hishuley Carmel sites. The pollen in the *gefet* most probably derived from the olives which were picked from trees growing on the western slopes of Mount Carmel or on the Kurkar ridges further to the south.

The function of the Hishuley Carmel structures may be assessed from the pollen spectra. The olive-poor samples of the Hishuley Carmel structures support the interpretation of their function as pits for pickling/drying whole olives intended for consumption, unlike the olive-oil extraction installations at Kfar Samir. Pickling may have been done while the fruits underwent debittering process by repetitive soaking in sea water. Such a process may have indeed resulted in washing off olive pollen grains that adhered to the oily skin of the fruits. This may explain the markedly low olive pollen in the Hishuley Carmel samples, as compared to fossil and recent *gefet* samples, where they are typically exceedingly high.

**References**

1. K. Faegri, J. Iversen, Textbook of Pollen Analysis, 4th ed. (John Willey and Sons, 1992).

2. M. Reille, Pollen et Spores d’Europe et d’Afrique du Nord, 2nd ed. (Laboratoire de Botanique Historique et Palynologie, Marseille, 1999).

3. E. Galili, M. Weinstein-Evron, Prehistory and paleoenvironments of submerged sites along the Carmel Coast of Israel. *Paléorient* **11**(1), 37-52; (1985).

4. W. Franke, Vitamin C in sea fennel (*Crithmun maritimum*), An edible wild plant. *Econ Bot* **36**, 163-165 (1982).

5. M. Weinstein-Evron, S. Chaim, Palynological investigations, in Linder E. and Kahanov Y. (eds.), The Maʽagan Mikhael Ship. The Recovery of a 2000-Year-Old Merchantman. (Israel Exploration Society, 2003), Vol. I. pp. 221-229.

6. E. Galili *et al.,* Atlit-Yam: A prehistoric site on the sea floor off the Israeli coast. *J Field Archaeol* **20**, 133-157 (1993).

7. E. Galili, D.J. Stanley, J. Sharvit, M. Weinstein-Evron, Evidence for earliest olive-oil production in submerged settlements off the Carmel Coast, Israel. *J Archaeol Sci* **24**, 1141-1150 (1997).

8. E. Galili, D. Cvikel, J. Benjamin *et al.,* Coastal paleoenvironments and prehistory of the submerged Pottery Neolithic settlement of Kfar Samir, Israel. *Paléorient* **44**(2), 113-132 (2018).

9. M. Weinstein-Evron, Biases in archaeological pollen assemblages: case studies from Israel. *AASP Contributions Series* **29**, 193-205 (1994).

Table 1: Pollen spectra of Hishuley Carmel (HC) structures A, B; Kfar Samir (KS) *gefet* (waste from olive oil extraction); recent *gefet* from Mount Carmel oil press; Submerged fossil clay paleosols from Kfar Samir (KS).


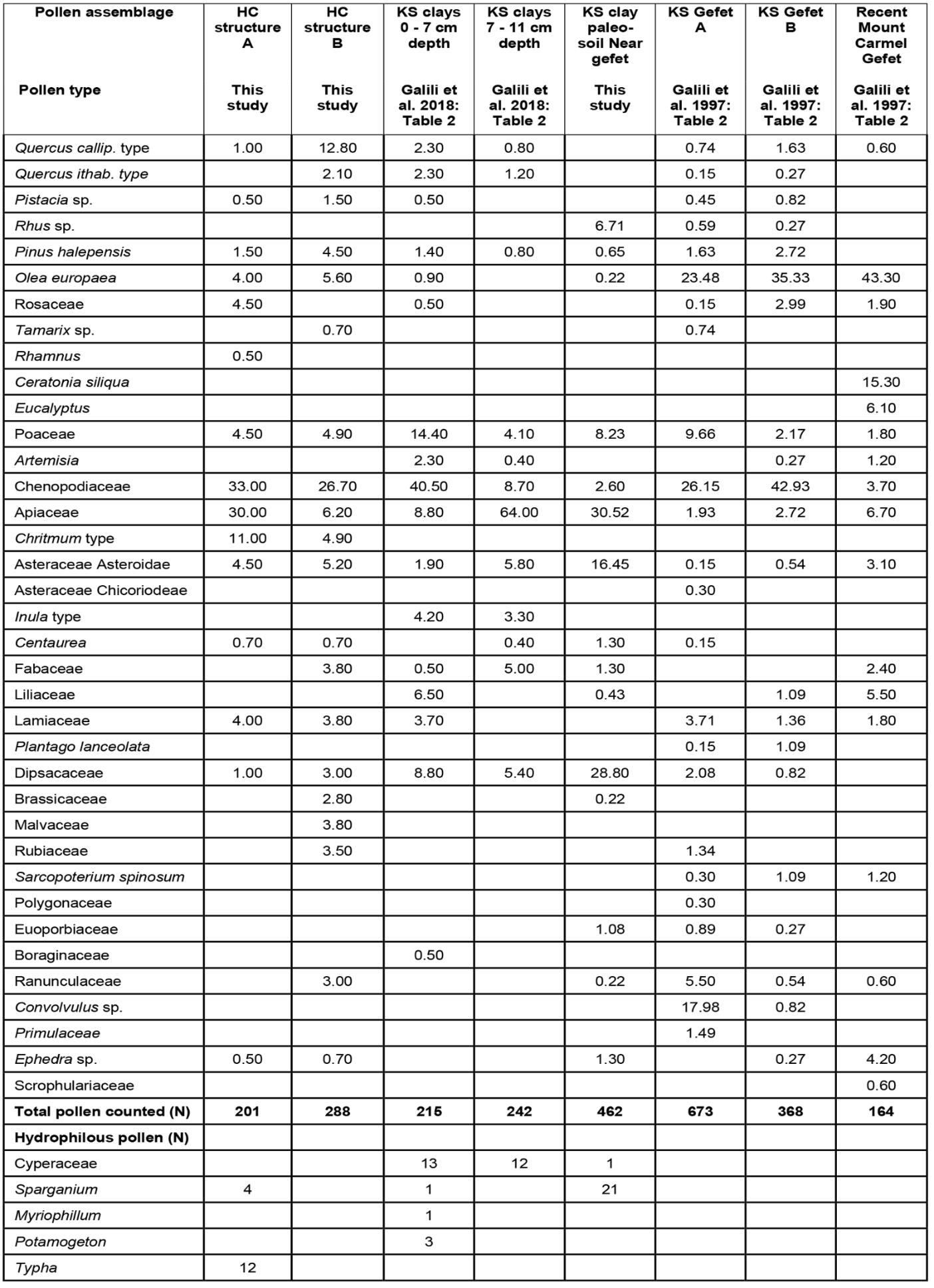


**SI Appendix 6: Experiment: The use of seawater in fermentation of olives**

Z. Ben-Barak-Zelas and A. Fishman

The protocol of E. Ramirez *et al.* for curing olives in brine, recommended in the Mediterranean traditional home production (Text ref. 60) was used. Three groups of Suri olives (Fig. 1) (200 fruits each) were used for the experiments. The fruits were picked from domesticated trees on Mount Carmel, west of Daliyat al-Karmel (32º 41’ 36.72’’ N; 35º 1’ 20.42’’ E) on 23.11.2019, after the first substantial rain of the season and when the olives were partly black. This follows the practice of local traditional farmers in the region [1]. October is the earliest date for picking olives for oil, some types are picked two weeks later. In another paper, Dag *et al.* [2] noted that table olives are picked earlier, i.e., in September. However, since it is not clear at which month the Hishuley Carmel olives were picked, it was assumed that they were harvested simultaneously with olives picked for oil. Thus, the pickling experiment was implemented with olives that were picked at the end of October, when olives for oil extraction are picked today. The fermentation was conducted by using the pickling system (protocol) proposed by Brennan (Text ref. 59), but in order to be closer to prehistoric practices, the soaking in a chemical was not applied and no starter was used. The experiment was conducted in three different treatments:

1.Three containers of olives fermented using sea water (~3% salt).

2. Three containers of olives fermented using sea water + 8% sea salt (final concentration ~11%).

3. Three containers of olives fermented using tap water + 11% NaCl (control).

For each treatment, 200 g of Suri olives were put in a plastic jar and the appropriate brine was added. Each treatment was done in triplicates without adding a starter culture (total 600 g for each treatment). To investigate the contribution of cutting/crushing which is important for removal of bitterness, each fermentation was also carried out with cut olives (one longitudinal cut with a knife) before placement in the jar. In total, we had six treatments in 18 jars (each treatment in triplicate) spontaneously fermenting at room temperature near a window facing south (Fig. 2). Microbial analysis was performed regularly on the brine for 112 days (Text Fig. 8). The pH was measured for each sample using a cyberscan pH-meter. Changes in olive flesh hardness during fermentation were monitored (puncture test) with a texture analyzer (Table 1). For the microbial analyses, 5 ml of the brine was taken periodically for analysis.

Fermentation of Suri olives was tested under three different conditions: (i) fermentation using seawater (~3% salt), (ii) fermentation using seawater+8% sea salt (final concentration ~11%), and (iii) fermentation using tap water+11% NaCl (control). In addition, a puncture test was conducted on a sample of each group after fermentation.


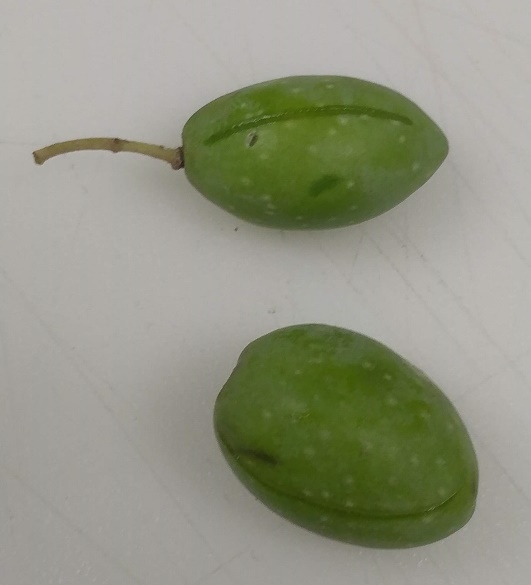


Fig. 1. Cut Suri olives before brining (Photo A. Fishman).

In total, there were six fermentation treatments in 18 plastic jars (each treatment in triplicate) spontaneously fermenting at room temperature near a window, facing south (Fig. 2).


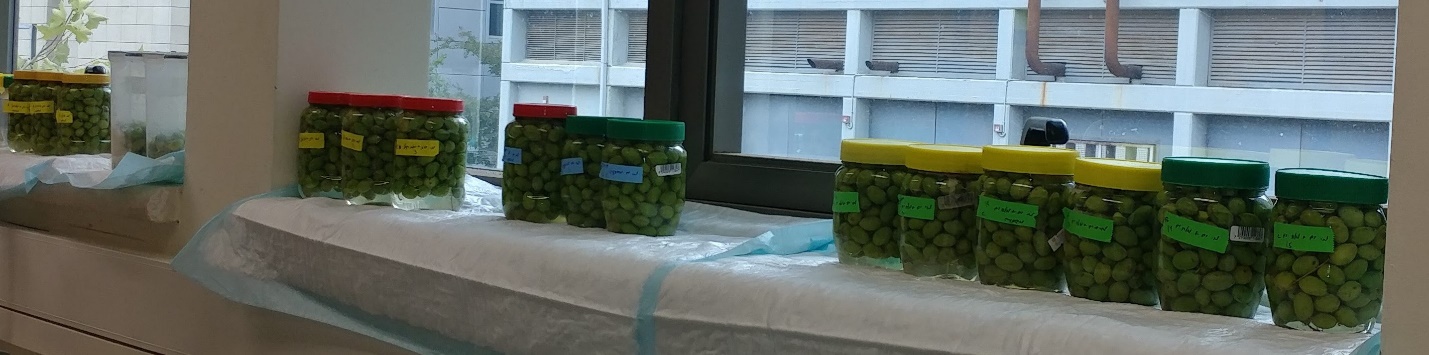


Fig. 2. The fermentation jars 5 days after the beginning of the experiment (Photo A. Fishman).

# Microbiological analysis

Microbial analysis was performed regularly on the brine at 0, 5, 13, 21, 34, 42 and 112 days of fermentation. A 5 ml sample was taken from each jar (Fig. 3). A dilution series of each sample was prepared from 10^-1^ to 10^-4^ in saline (0.85% NaCl) and these were plated using the drop method. Four drops of 10-20 µl from each dilution were plated onto Nutrient Agar + Cycloheximide 25mg/L (Sigma) for estimation of total aerobic bacteria. The enumeration of total yeast and mould was carried out in the selective medium, Oxytetracycline-glucose-yeast extract agar containing 100 mg/L Oxytetracycline. The plates were incubated at 30°C for 48h. To identify Enterobacteriaceae, 1 ml sample (diluted and non-diluted) was plated onto **Violet Red Bile Agar** with the pour-plate method. The plates were incubated at 37°C for 24h.

# For identification of lactic acid bacteria (LAB) we used YE + calcium carbonate agar plates (in w/v: 0.5% peptone, 2% Glucose, 0.5% yeast extract, 1.5% agar, 0.3% CaCO_3_). These agar plates are turbid because the solubility of the calcium carbonate is very low. As LAB grow, they produce lactic acid, which lowers the pH of the media. This in turn increases the solubility of calcium carbonate. Therefore, as the calcium carbonate dissolves, that particular area becomes clear indicating growth of the colony. A suspicious colony is analyzed for catalase activity using 3% hydrogen peroxide. LAB are negative in the catalase activity test.

**Puncture test**

From each treatment at least nine olives were taken. A slice of the olive flesh with dimensions of 10 mm diameter and 5 mm height was cut. The samples were penetrated by a cylinder probe to 50% of the initial height at constant speed of 2.5 mm/min with a 50 N load transducer. Puncture determinations were carried out at 0, 30 and 60 days.

**Results**

# Microbial analysis: For the microbial analyses, 5 ml of the brine was taken periodically. With the fermentation progress, the brine became turbid suggesting an increase in microbial growth (Fig. 3).


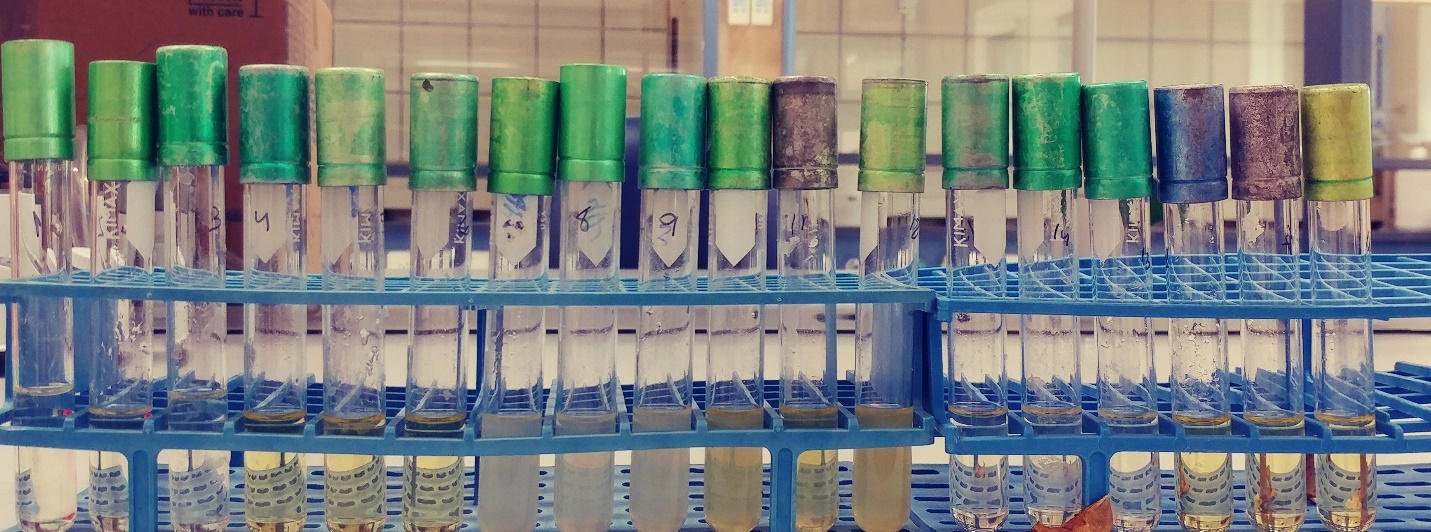


Cut

Uncut

Cut

Uncut

Cut

Uncut

Fermentation using tap water+11% NaCl

Fermentation using seawater

Fermentation using seawater+8% sea salt

A


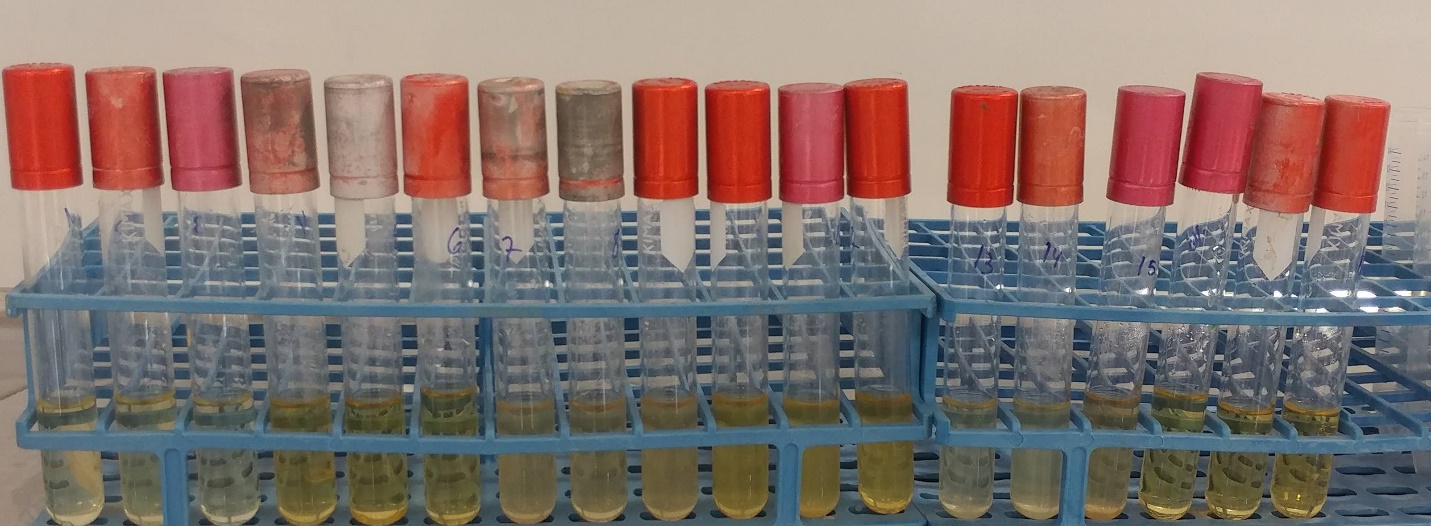


B

Fig. 3. Brine for microbiological analysis. A - after 13 days; B – after 42 days. Tube order is the same in both pictures (Photo A. Fishman)

It is visible in Figure 3, that the fermentation liquid of cut olives was more darkly colored than in the uncut olives. Following the cut in the olive flesh, a cascade of enzymatic reactions began causing brown spots due to melanoid formation. The dark pigment in the brine resulted from diffusion from the olives.

The total aerobic bacteria counts in the seawater fermentation after 42 days were 3×10^3^ CFU/ml and 2×10^5^ CFU/ml in the uncut and cut olives, respectively. At the end of fermentation (after 112 days), the counts were similar, 2.1×10^2^ CFU/ml and 3.76×10^3^ CFU/ml in the uncut and cut samples, respectively. Fermentation with seawater+8% sea salt led to 4×10^4^ CFU/ml in the uncut olives. However, in the control, fermentation with tap water+11% NaCl, and fermentation with seawater+8% sea salt of cut olives samples, we did not identify aerobic bacteria (Text Fig. 8b in text). Only yeast cells grew on the YE + calcium carbonate agar plates, but not LAB colonies as was expected. We plated known LAB cells as a control and they grew well. The growth of Enterobacteriaceae bacteria were observed at the beginning of the fermentation process (first 21 days) only in seawater brine with cut olives and in one jar of seawater+8% sea salt. The count was 3.23 log (CFU/ml) in maximum. With the progress of the fermentation and the decline in the pH, the Enterobacteria were eliminated as expected [1]. Counts of yeast and mold were executed at time 0 and after 21, 34, 42 and 112 days (Text table 2).

There are no significant differences between the samples. In all samples, yeast and mold were identified. In a few jars, especially from fermentation with seawater+ 8% sea salt, mold was observed in the upper layer of the brine (Fig. 4).

| 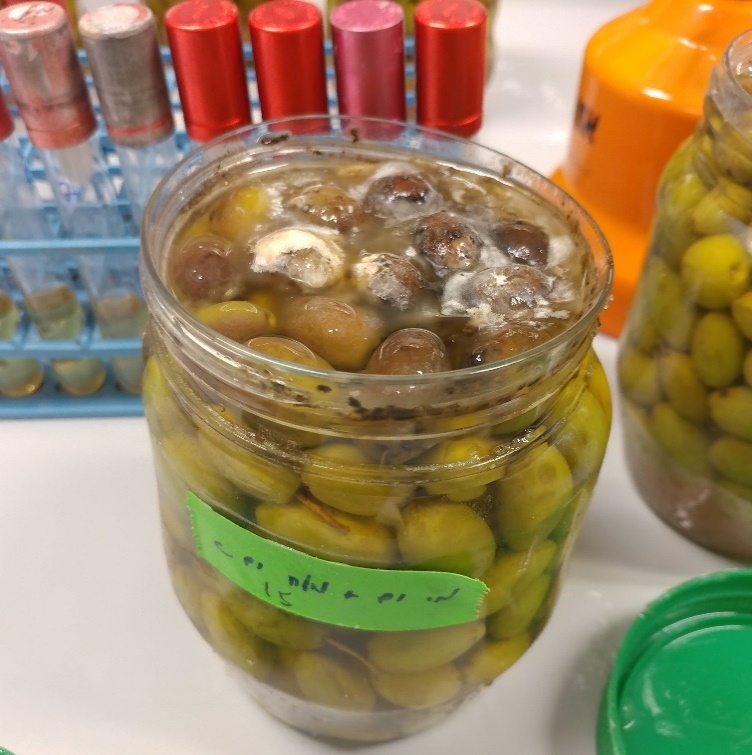 | 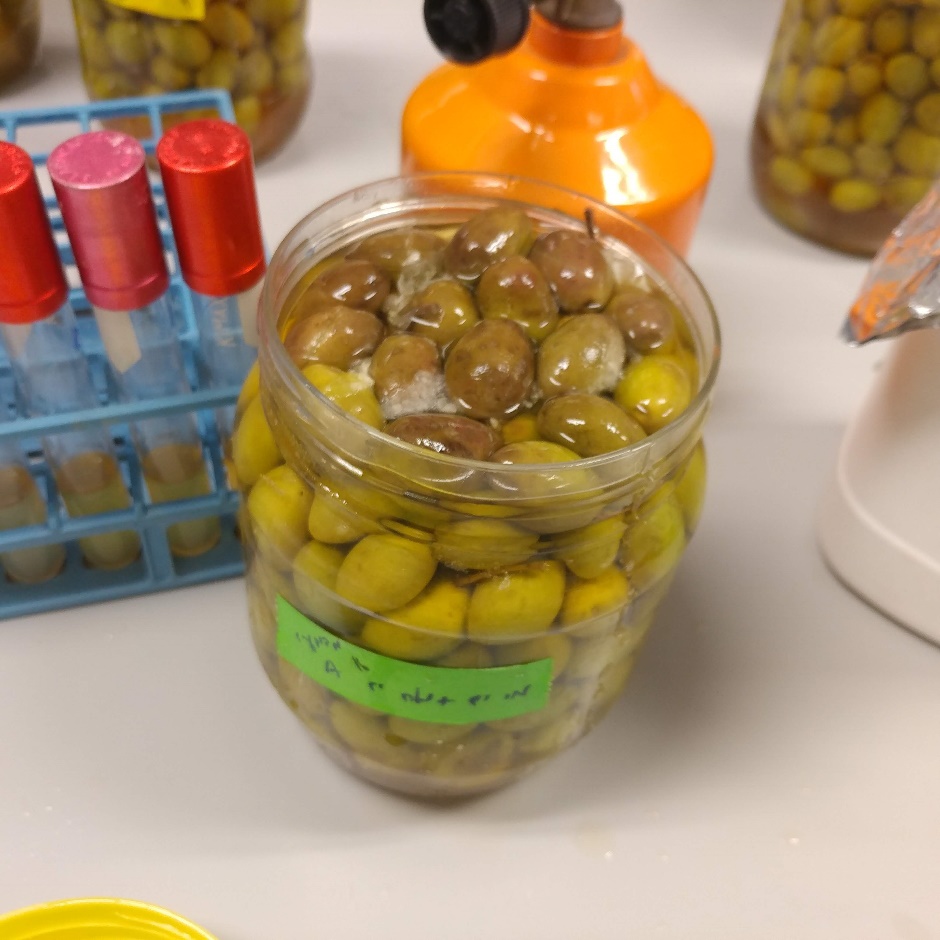 | 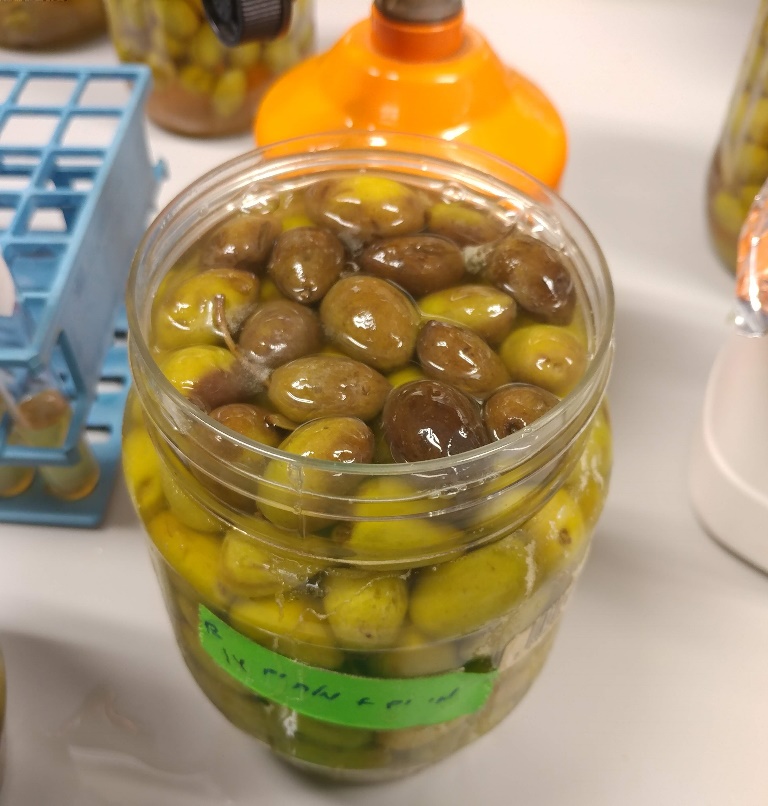 |
| --- | --- | --- |

Fig. 4. Mold in the upper layer of the brine seawater+ 8% sea salt (Photo A. Fishman)

**Puncture test:** The consistency of the olives was monitored with a texture analyzer. In order to learn about changes in olive flesh elasticity between the different treatments and the initial state, we compared a parameter called **Young's Modulus**. It defines the relationship between stress (Force to area) and deformation in a solid material. The larger the Young's Modulus, the less elastic the material is; a greater force is required to cause deformation. Another parameter is the stiffness of the olive. The results are presented in Table 1 and in the text Fig. 8a. After 30 days there is no correlation between the brine, the cutting of the olive fruit and the puncture test parameters (Young's Modulus, Stiffness) compared to the initial values, before the fermentation. However, after two months the values appear to be similar across all the treatments. It can be seen more visibly in Fig. 8c in the text.

Table 1: Puncture test results in fresh and fermented olives in the different treatments.

|  | **Time 0** | **After 30 days** | | | | | |
| --- | --- | --- | --- | --- | --- | --- | --- |
|  |  | **Tap water + 11% NaCl** | | **Seawater** | | **Sea water+ 8% sea salt** | |
|  |  | **Uncut** | **Cut** | **Uncut** | **Cut** | **Uncut** | **Cut** |
| **Stiffness (N/m)** | 27,211 ± 4,451 | 32,103 ± 10,208 | 10,971 ± 5,408 | 23,982 ± 6,927 | 30,529 ± 8,683 | 77,471 ± 16,542 | 22,808 ± 3,932 |
| **Young's Modulus (MPa)** | 1.7 ± 0.3 | 2.0 ± 0.6 | 0.7 ± 0.3 | 1.6 ± 0.4 | 1.9 ± 0.5 | 5 ± 1 | 1.4 ± 0.2 |
| **After 60 days** | | | | | | | |
|  | | **Tap water + 11% NaCl** | | **Seawater** | | **Seawater+ 8% sea salt** | |
|  |  | **Uncut** | **Cut** | **Uncut** | **Cut** | **Uncut** | **Cut** |
| **Stiffness (N/m)** | | 48,863± 1,292 | 34,647± 3,899 | 32,967 ± 7,573 | 42,529 ± 8,736 | 25,642 ± 2,067 | 37,718 ± 4,509 |
| **Young's Modulus (MPa)** | | 3.11 ± 0.08 | 2.2 ± 0.2 | 2.0 ± 0.5 | 2.7 ± 0.5 | 1.6 ± 0.1 | 2.4 ± 0.3 |

**Discussion**

Fermentation of table olives is not a fully predictable process. The microbiota of olives is different depending on the olive strain, cultivar and the type of fermentation (with or without alkali treatment etc.) [3, 4]. Thus, the results in the literature vary depending on the aspects mentioned above. We saw higher bacterial counts in fermentation with sea water than the control with tap water+11% NaCl. It may be due to the lower concentration of the salt which allows the bacteria to thrive. The uncut olive with sea water+8%NaCl had high bacterial counts than the cut olives in the same brine. This may be due to spoilage not associated with brine since the cut is supposed to affect the taste of the olive. In all fermentations, we saw similar counts of yeast. According to Heperkan [3], yeast have a significant role in fermentation of olives together with LAB [3]. Similar to our results, in previously reported papers LAB was not observed in some types of natural green olives. It might be because LAB are moderately inhibited, indirectly, due to the presence of phenolic compounds. Therefore, yeasts become the majority [3]. The viable counts that were reported in the literature were similar to the counts observed here.

In the puncture test, after 30 days of fermentation we did not see a clear trend in the results. The Young's Modulus according to Fadda *et al.* [5] which measured the influence of different sodium chloride brine concentrations on texture profiles after 30 days there is no significance different. Whereas after 60 days they managed to see a significant difference; The olives processed with higher NaCl concentrations (7% compare to 4%) showed higher flesh stiffness [5]. We did not see differences between the treatments after 60 days. We used a Suri olive which might be different from the olives that were used by Fadda *et al.* [5] but they didn’t specify what type they used. Another study did not find differences in puncture test parameters after fermentation of black olives using different NaCl concentration (40,60,80 g/L) [6]. Therefore, it appears that the type of the olive has a major influence on the results.

**Conclusions**

Since the use of seawater in olive fermentation gave similar values as the other brines, it seems that seawater can successfully be used for olive fermentation.

**References**

1. A. Dag, G. Harlev, S. Lavee, I. Zipori, Z. Kerem, Optimizing olive harvest time under hot climatic conditions of Jordan Valley, Israel. *Eur J Lipid Sci Technol* **116**, 169-176 (2014).

2. A. Dag, A. Bustan, A. Avni, I. Tzipori, S. Lavee, J. Riov, Timing of fruit removal affects concurrent vegetative growth and subsequent return bloom and yield in olive (*Olea europaea* L.). *Sci Hort* **123**, 469-472 (2010).

3. D. Heperkan, Microbiota of table olive fermentations and criteria of selection for their use as starters. *Front Microbiol* **4**, 1-11 (2013).

4. H. Lucena-Padrós, JL. Ruiz-Barba, Microbial biogeography of Spanish-style green olive fermentations in the province of Seville, Spain. *Food Microbiol* **82**, 259-268 (2019).

5. C. Fadda, A. Del Caro, AM. Sanguinetti, A. Piga, Texture and antioxidant evolution of naturally green table olives as affected by different sodium chloride brine concentrations. *Grasas y Aceites* **65** (2014).

6. CMM. Coelho, C. de M. Bellato, JCP. Santos, EMM. Ortega, SM. Tsai, Effect of phytate and storage conditions on the development of the ‘ hard-to-cook.’ *J Sci Food Agric* **1243**, 1237-1243 (2007).

**SI Appendix 7: Pilot experiment of dry salting of olives picked from naturally growing trees on the Atlit ridge**

E. Galili

The naturally growing olives trees which grow on the Atlit kurkar ridge some 500 m inland from the coast and 10 km south of Hishuley Carmel, are suspected to be wild population. Their fruits are relatively small, with a low percentage of oil (7.18%), determined in fruit paste by NIR instrument – Oliv Scan, FOSS [1] compare to typical rained orchards which have more than 20% in their fruit in general (Oz Barazani and Arnon Dag personal communication). This indicates that they actually have more similarity to wild population than to domesticated one. Given their location in the Carmel coast adjacent to the sea, they may represent the ancestor used for picking olive that were used in Hishuley Carmel site. In accordance, we undertook a pilot study aimed at checking if they can be dry salted to be consumed as table olives.

The following, local traditional protocol was applied:

1) About 800 gr of olives were picked from the naturally growing trees during January, when they were blackened and ripe and many of them already fallen from the tree (Figs. 1, 2).

2) The fruits were washed with fresh water.

3) Boiling fresh water was poured on the olives and they were soaked in the hot water until the water cooled to ambient temperature.

4) The olives were placed on a flat wooden tray and covered with a 1 cm layer of thick, coarse sea salt. The tray was placed in an open shaded location outdoor at ambient temperature for 10 days.

5) After 10 days, the olives were mixed in with the salt and left to dry for another 10 days (Fig. 3).

6) After 20 days of dehydration, the olives were mixed with a little olive oil and were placed in a sealed glass jar.

The olives were kept in the glass jar with no refrigeration, and were consumed within two months.

Their test was somewhat bitter, but they were proved to be tasty.


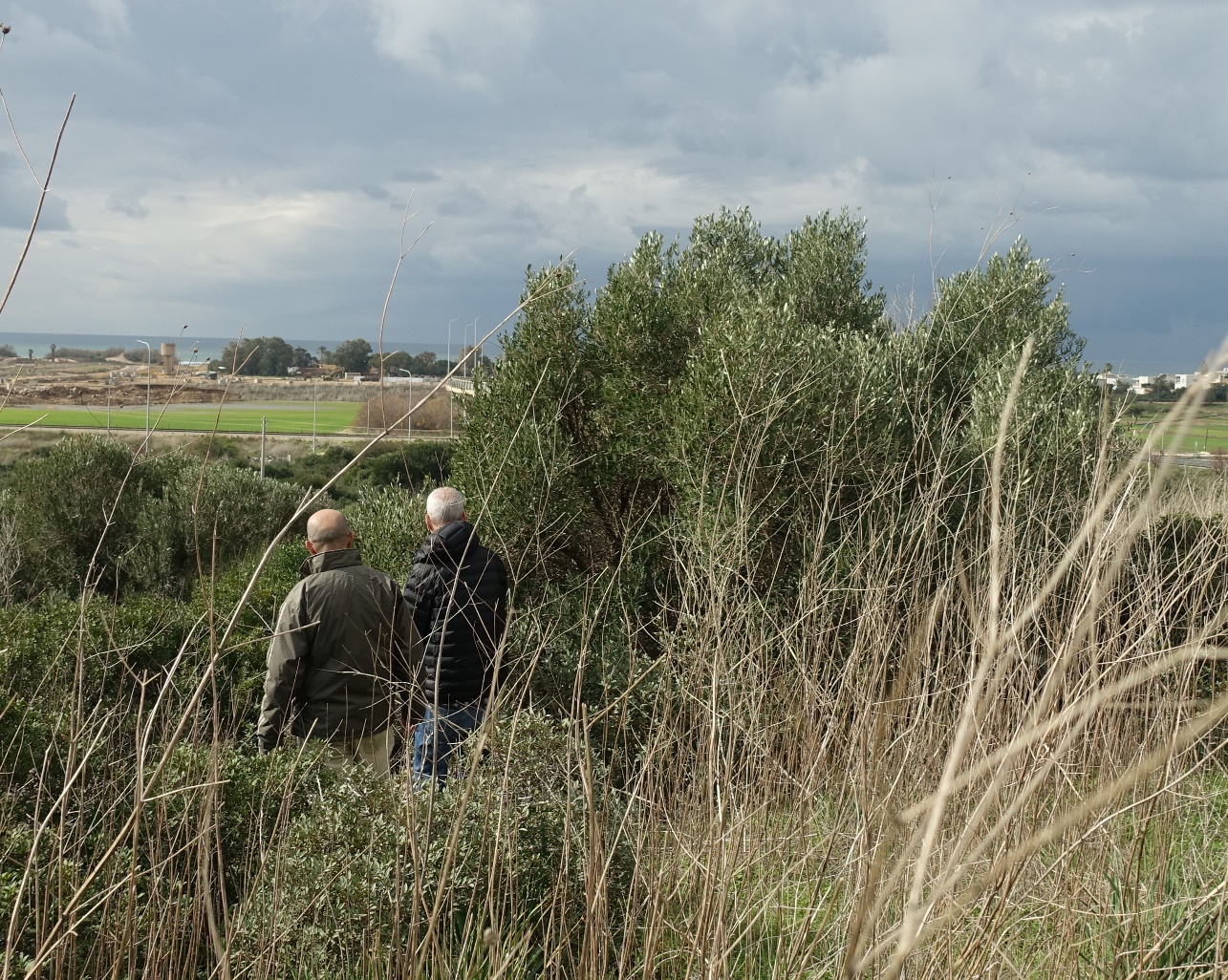


Fig. 1. Picking olives from naturally growing tree south of Atlit (Photo E. Galili January 2020).


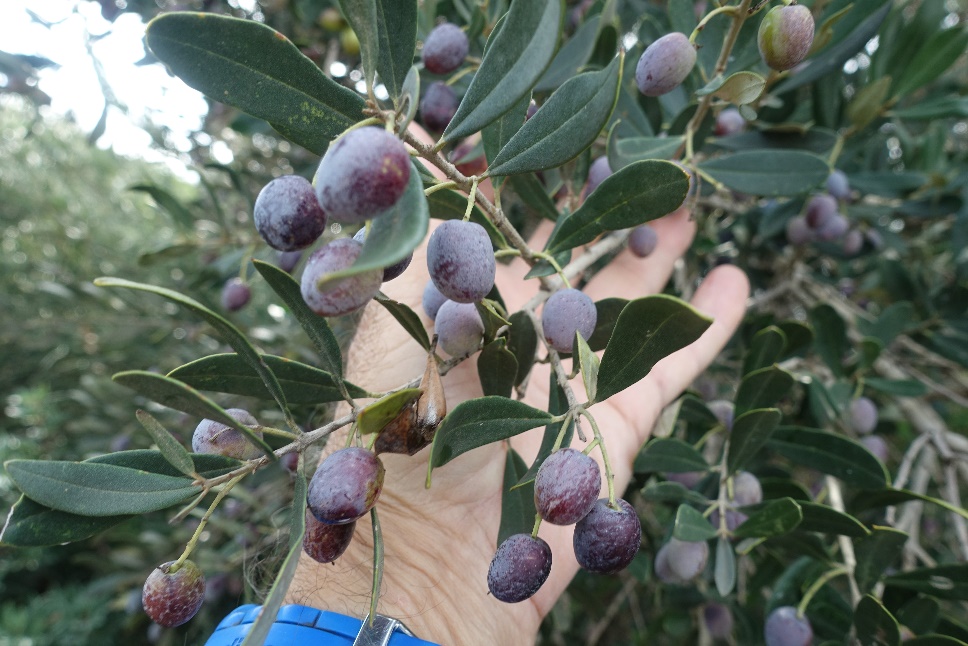


Fig. 2. The olives on the naturally growing tree before picking. (Photo E. Galili January 2020).


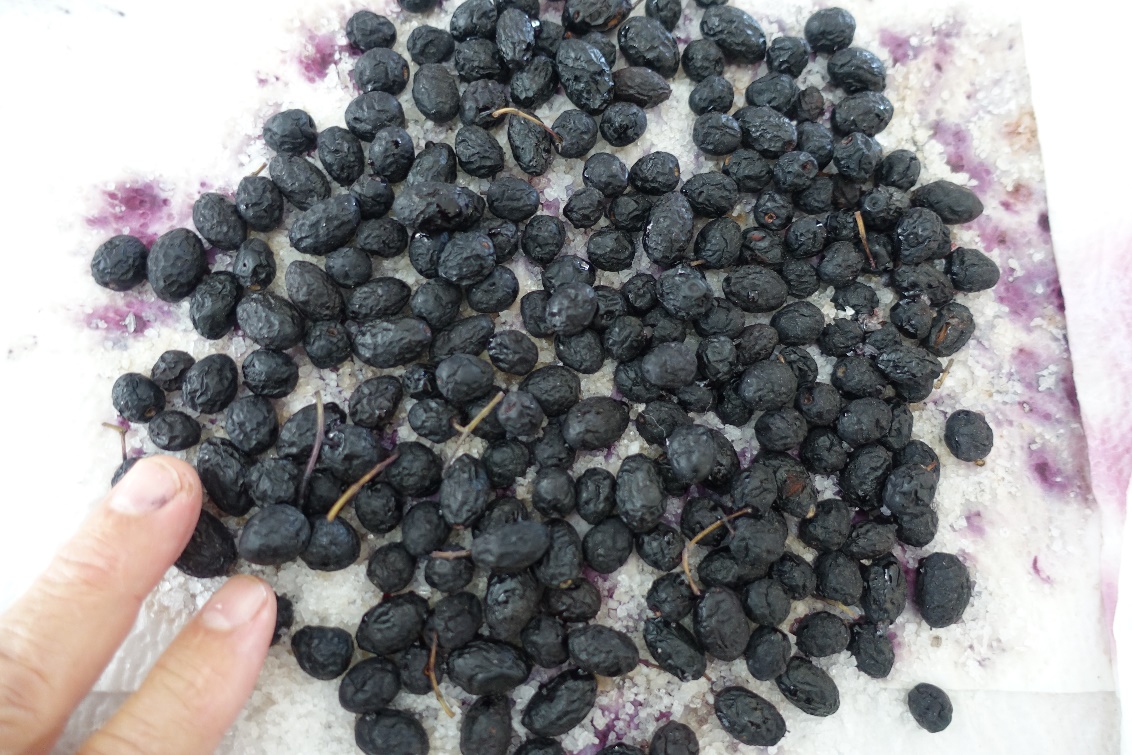


Fig 3. The dry salted olives after 10 days (Photo E. Galili January 2020).

**Reference**

1. I. Zipori, A. Bustan, Z. Kerem, A. Dag, Olive paste oil content on a dry weight basis (OPDW): an indicator for optimal harvesting time in modern olive orchards. *Grasas y Aceites* **67** (2), 137 (2016).

**SI Appendix 8: Holocene sea level changes and coastal changes in the Carmel coast**

E. Galili

Submerged Neolithic settlements that were inundated following global sea-level rise during the Holocene were discovered along the northern Carmel coast (See SI Appendix 1 above). Archaeological analyses of these settlements, coupled with other archaeological and geomorphological sea-level markers (e.g., deposits of coastal marshes, abrasion platforms, wave notches and vermetidae colonies) have enabled us to reconstruct a tentative curve representing the sea-level changes in the Carmel coast (Fig. 1). There is a correlation between water depth, location offshore and site age: the older the sites, the deeper and the further offshore they are. This suggests a direct correlation between sea-level rise and the abandonment of coastal settlements and their translocation eastwards. The earliest recorded submerged site, Atlit-Yam (dated to 9100 to 8500 BP) is located 200–400m offshore, at a depth of -8–12m. When the site was occupied, sea level was ca. 16 m lower than today and the coastline was ca. 1000 m offshore. It was rocky and formed a laguna, (Fig. 2 left). Fifteen other inundated settlements are dated to the late Pottery Neolithic/Chalcolithic period (some 7500-6500 BP). These sites are presently located 1–200m offshore at 0–5m below sea level. At the time they were occupied, sea level was ca. 7 m lower than today and the coastline was a few hundred metres off the present coastline. A row of elongated islets stretched a few hundred meters off the Pottery Neolithic coastline, which was straight and sandy (Fig. 2 center) [1-3].


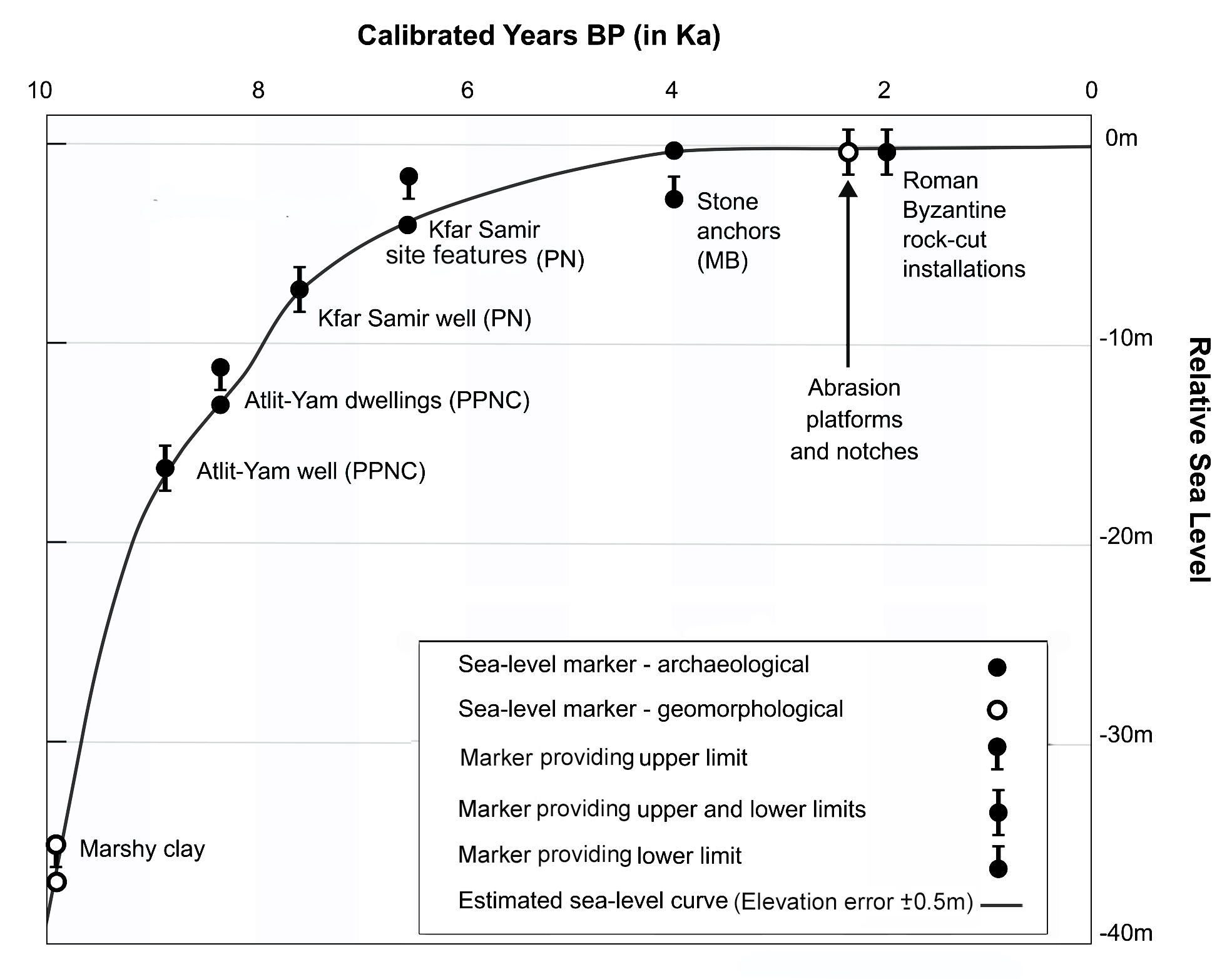


Fig. 1. A curve depicting the sea-level changes in the Carmel coast during the last 10,000 years.

The data derives from archaeological finds and geological features [Modified after refs. 1- 3].


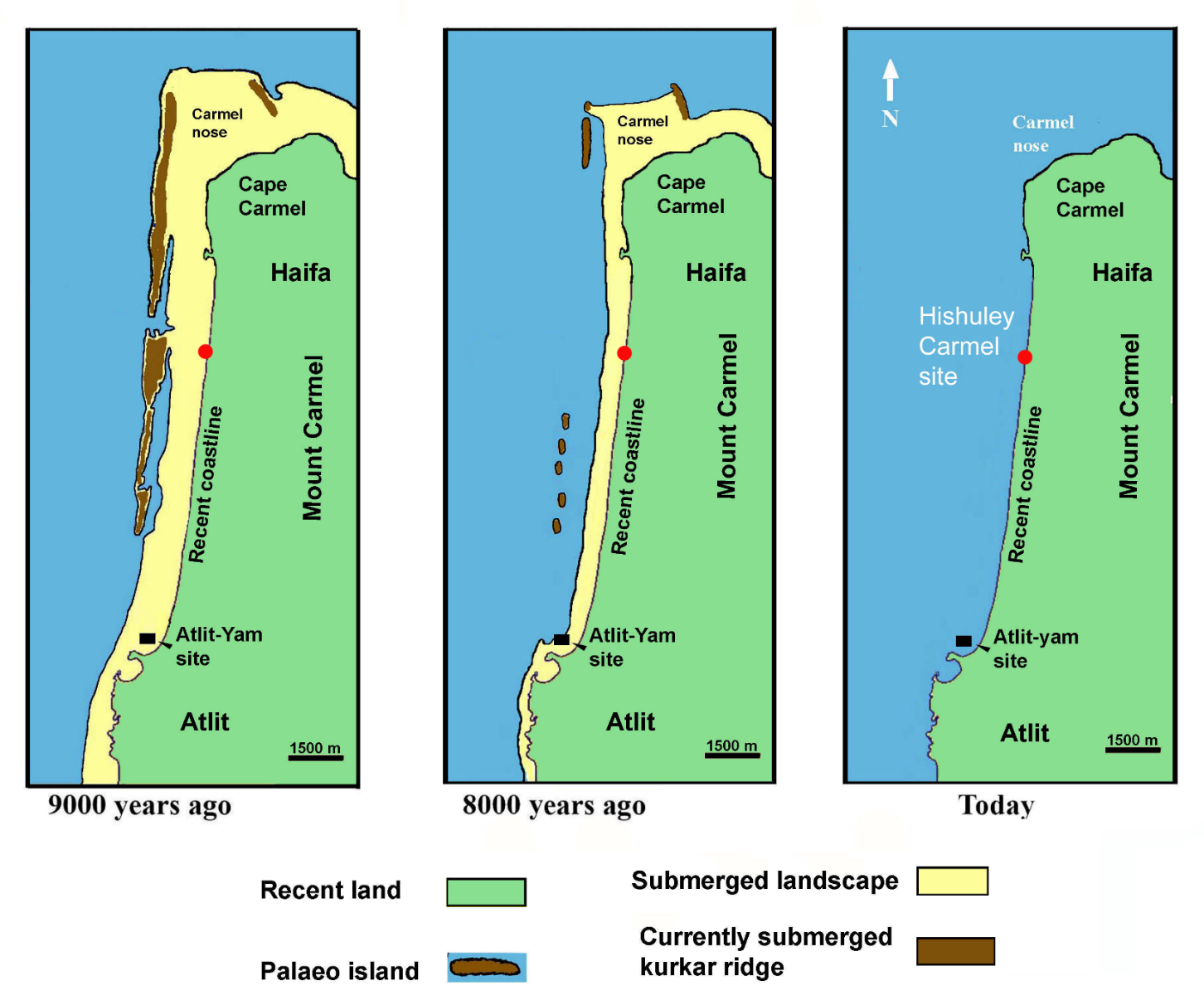


Fig. 2. Coastal changes in the northern Carmel coast during the last 9,000 years [Modified after ref. 1].

**References**

1. E. Galili, B. Rosen, M. Weinstein-Evron, I. Hershkovitz, V. Eshed, L.K. Horwitz, Israel: Submerged Prehistoric Sites and Settlements on the Mediterranean Coastline — the Current State of the Art, in: Bailey, G. *The Archaeology of Europe’s Drowned Landscapes*. Springer Nature: 443-481 (2000).

2. E. Galili, D. Zviely, M. Weinstein-Evron, Holocene sea-level changes and landscape evolution on the northern Carmel coast (Israel). *Mediterranée* **1** (2), 1-8 (2005).

3. E. Galili, J. Benjamin, V. Eshed, B. Rosen, J. McCarthy, L.K. Horwitz, A submerged 7000-year-old village and seawall demonstrate earliest known coastal defence against sea-level rise. *PLoS One* **14**, e0222560 (2020).
